# Post-marketing safety assessment of epinephrine: an analysis of the US FDA adverse event reporting system

**DOI:** 10.3389/fmed.2025.1727631

**Published:** 2025-12-18

**Authors:** Binglin Yan, Qing Xiao, Yipeng Jiang

**Affiliations:** 1 Department of Emergency Medicine, The 971st Hospital of the People’s Liberation Army Navy, Qingdao, China; 2 Department of Emergency Medicine, The 8th People’s Hospital of Qingdao, Qingdao, China; 3 Department of Urology, The 971st Hospital of the People’s Liberation Army Navy, Qingdao, China

**Keywords:** adverse events, data mining, epinephrine, FAERS, pharmacovigilance

## Abstract

**Objective:**

Epinephrine, a sympathomimetic catecholamine, is extensively employed for the treatment of anaphylaxis. This research evaluates adverse events (AEs) associated with epinephrine, utilizing data from the US FDA Adverse Event Reporting System.

**Methods:**

AE reports related to epinephrine submitted from Q1 2004 to Q4 2024 were extracted for analysis. Multiple signal detection methodologies were employed, including Reporting Odds Ratio (ROR), Proportional Reporting Ratio (PRR), Bayesian Confidence Propagation Neural Network (BCPNN), and Empirical Bayes Geometric Mean (EBGM).

**Results:**

Out of 9,262 reports identifying epinephrine as the primary suspected medication, 24 system organ classes (SOCs) and 264 significant preferred terms (PTs) were recognized. General disorders and administration site conditions constituted the most common SOC (*n* = 6,112). At the PT level, drug ineffective was predominant (*n* = 1,867), whereas injection site ischemia demonstrated the strongest signal (ROR: 3242.28, PRR: 3236.49, IC: 10.43, EBGM: 1380.84). Additionally, several notable AEs not mentioned in current drug labeling exhibited substantial signals, such as myocardial stunning, systolic anterior motion of mitral valve, left ventricle outflow tract obstruction, harlequin syndrome, injection site nerve damage, and injection site movement impairment. The median interval to AE onset was 0 day (interquartile range [IQR] 0–0 day) with most of cases emerged within 24 h after application of epinephrine.

**Conclusion:**

This investigation identified numerous previously unreported AE signals associated with epinephrine. Further clinical studies are necessary to substantiate these findings and elucidate the causal relationships.

## 1 Introduction

Anaphylaxis is a severe, rapidly developing systemic allergic response that poses a potential threat to life. It primarily manifests clinically through dermatological symptoms such as urticaria, facial or lip edema, skin redness, and itching (1). There is a global increase in rates of all-cause anaphylaxis, with an estimation of lifetime prevalence between 1.6 and 5.1% (2, 3). Food allergy represents one of the primary triggers of anaphylaxis leading to emergency department visits in the United States, accounting for approximately 30,000 cases annually (4). According to Patel et al., the combined direct medical expenses and indirect costs associated with food allergies and anaphylactic reactions are estimated at $340 million per year (5). Considering the treatment of anaphylaxis, epinephrine, antihistamine agents, and glucocorticoids were the first-line, second-line, and adjunctive therapy, respectively (6).

Epinephrine is a sympathomimetic catecholamine, which non-selectively activates both alpha- and beta-adrenergic receptors. It induces peripheral vasoconstriction, increases myocardial contractility and heart rate, relaxes bronchial smooth muscles, which is indicated in the treatment of anaphylaxis, cardiac arrest, severe asthma, and hypotensive shock (7). Pallor, tremor, anxiety, weakness, dizziness, sweating, palpitations, and arrhythmias were common adverse reactions of epinephrine (8). Although epinephrine has been used in clinical practice for decades, a comprehensive and detailed safety evaluation remains essential. Given its frequent application, even rare adverse effects that have not yet been clearly identified could affect a large number of patients.

The FDA Adverse Event Reporting System (FAERS) offers a pharmacovigilance repository that compiles drug safety surveillance data from real-world clinical scenarios. FAERS enables a rigorous assessment of epinephrine’s safety profile through detailed analysis of adverse drug reactions. To date, there has been no specific research that leverages FAERS data for a comprehensive exploration of epinephrine’s safety characteristics. Therefore, this study systematically examines adverse events (AEs) associated with epinephrine through various signal detection methodologies, thereby providing robust, evidence-based insights into its clinical risk assessment.

## 2 Methods

### 2.1 Data sources

To align with the drug’s approval timeline, this study selected data from the FAERS database spanning from the first quarter of 2004 to the fourth quarter of 2024. Data processing was carried out using R software (version 4.3). The FAERS database contains 7 key data tables: demographic and administrative information (DEMO), report sources, drug information (DRUG), indications for use, drug therapy start and end dates, adverse events, and patient outcomes.

### 2.2 Data extraction

**Figure 1:**
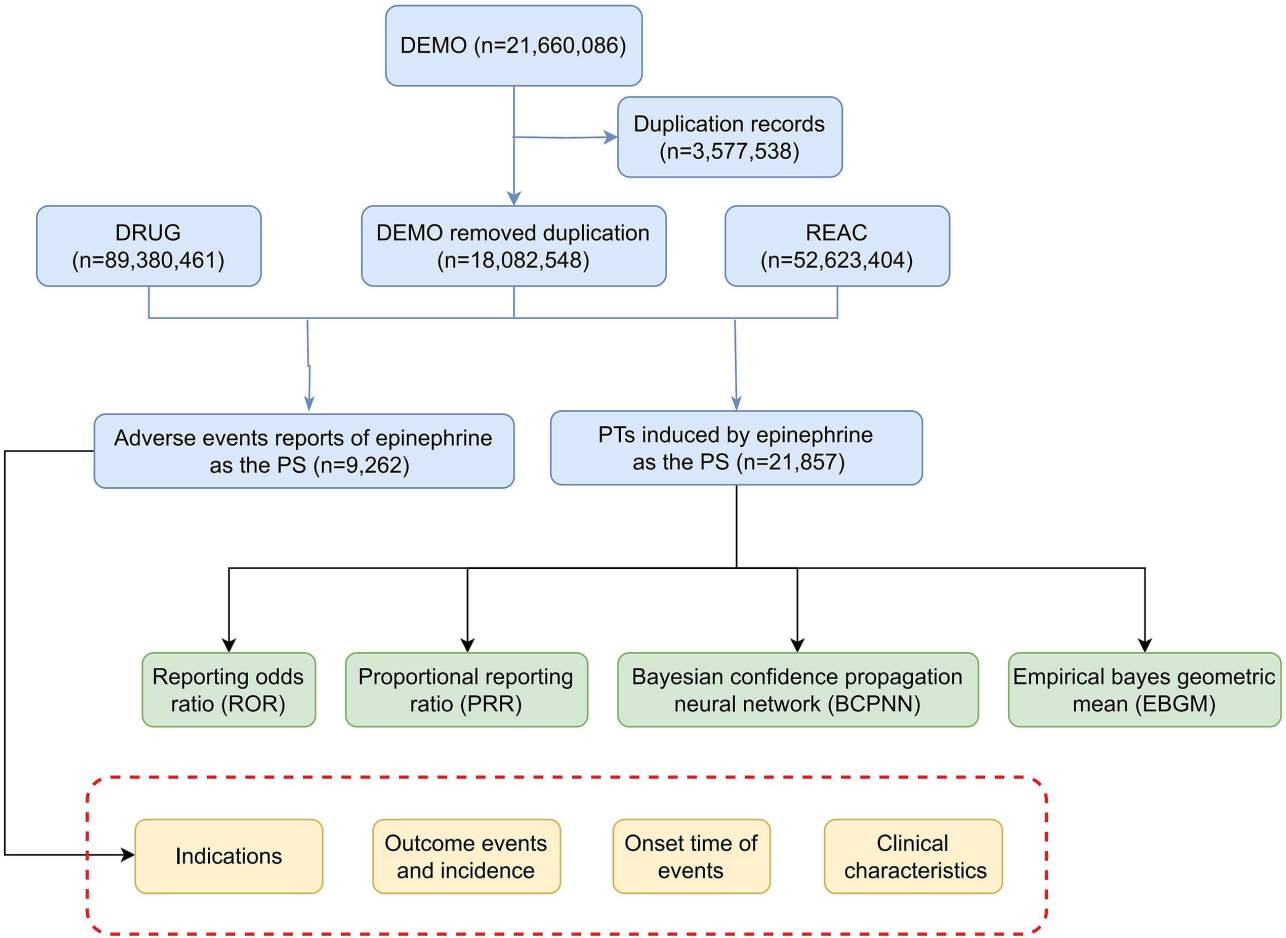
The flow diagram of selecting epinephrine-related adverse events from FDA adverse event reporting system database. PS, primary suspect; PT, preferred term.

Following FDA recommendations for eliminating duplicate reports, we identified the fields PRIMARYID, CASEID, and FDA_DT from the DEMO table for filtering. If multiple reports had the same CASEID, we retained the one with the most recent FDA_DT. In cases where both CASEID and FDA_DT matched, the report with the highest PRIMARYID was selected. To standardize drug names, we used Medex_UIMA_1.3.8. For data extraction from the DRUG file, we focused on the generic drug name “epinephrine,” “adrenaline” and its brand name “epipen” retrieving adverse event reports where epinephrine was identified as the primary suspected drug. We also collected clinical details such as patient age, gender, reporter information, region, reporting date, and the outcomes associated with epinephrine-related adverse events (AEs). The time-to-onset for AEs, which is the interval between the initiation of epinephrine therapy and the onset of the AE, was evaluated by excluding reports with missing, ambiguous, or erroneous date information (as shown in Figure 1).

### 2.3 Signal categorization and filtering

Adverse events were categorized according to the preferred term (PT) and system organ class (SOC) in the Medical Dictionary for Regulatory Activities version 26.1. The analysis focused on PTs that appeared in at least three AE reports within the FAERS database.

### 2.4 Statistical analysis

Descriptive statistics were employed to characterize the epinephrine-associated AE reports. To identify potential safety signals between epinephrine and AEs, four disproportionality analysis techniques were applied: the Reporting Odds Ratio (ROR), Proportional Reporting Ratio (PRR), Bayesian Confidence Propagation Neural Network (BCPNN), and Empirical Bayes Geometric Mean (EBGM). Detailed algorithms and criteria for these methods are outlined in Supplementary Tables S1, S2. Only those AE signals that met the criteria across all four algorithms were selected, allowing for validation from multiple perspectives to ensure the reliable detection of safety signals.

## 3 Results

### 3.1 Basic information

A total of 18,082,548 AE reports were obtained from the FAERS database between the first quarter of 2004 and the fourth quarter of 2024, and the epinephrine was the primary suspected drug of the AE in 9,262 reports. The proportion of female was higher than that of male (50.29% vs. 32.39%). The largest percentage of reports came from patients aged 18–44 years (17.56%), subsequently those aged 45–64 years (16.32%) and under 18 years (11.97%). The largest proportion of AE reports came from consumers (44.57%), while medical experts submitted the majority (49.73%), including pharmacists (20.09%), physicians (16.71%), other healthcare professionals (12.90%), and registered nurses (0.03%). The majority of AEs was reported in the United States (61.27%), followed by Canada (4.73%), United Kingdom (2.61%), Japan (2.44%), and Australia (1.75%). The number of reports reached its peaks in 2017 (14.05%) and 2021 (10.94%). Epinephrine was administrated intramuscularly in highest percentage of AE reports (15.17%), subsequently through intravenous (IV) and subcutaneous (SC) route (6.72 and 3.35% respectively). Initial or prolonged hospitalization was the most common severe outcome (25.96%) and anaphylactic reaction was the most reported indication, accounting for 16.01% (Table 1).

**Table 1:** Basic information of adverse event reports related to epinephrine in FDA adverse event reporting system database (2004 Q1-2024 Q4).

Factors	Number of events (%)
Gender	
Female	4,658 (50.29)
Male	3,000 (32.39)
Unknown	1,604 (17.32)
Age	
<18	1,109 (11.97)
≥18, <45	1,626 (17.56)
≥45, <65	1,512 (16.32)
≥65, <75	603 (6.51)
≥75	374 (4.04)
Unknown	4,038 (43.60)
Reporter	
Consumer	4,128 (44.57)
Pharmacist	1,861 (20.09)
Physician	1,548 (16.71)
Other health-professional	1,195 (12.90)
Lawyer	16 (0.17)
Registered nurse	3 (0.03)
Unknown	511 (5.52)
Reported countries	
United States	5,675 (61.27)
Canada	438 (4.73)
United Kingdom	242 (2.61)
Japan	226 (2.44)
Australia	162 (1.75)
Others	2,519 (27.20)
Report year	
2004	53 (0.57)
2005	50 (0.54)
2006	87 (0.94)
2007	94 (1.01)
2008	243 (2.62)
2009	131 (1.41)
2010	222 (2.40)
2011	203 (2.19)
2012	225 (2.43)
2013	252 (2.72)
2014	416 (4.49)
2015	579 (6.25)
2016	851 (9.19)
2017	1,301 (14.05)
2018	696 (7.51)
2019	660 (7.13)
2020	680 (7.34)
2021	1,013 (10.94)
2022	462 (4.99)
2023	534 (5.77)
2024	510 (5.51)
Route of administration	
Intramuscular	1,405 (15.17)
Intravenous	622 (6.72)
Subcutaneous	310 (3.35)
Others	6,925 (74.77)
Serious outcomes	
Hospitalization—initial or prolonged	2,109 (25.96)
Life-threatening	1,455 (17.91)
Death	753 (9.27)
Required intervention to prevent permanent impairment/damage	104 (1.28)
Disability	74 (0.91)
Congenital anomaly	8 (0.10)
Others	3,620 (44.56)
Indications	
Anaphylactic reaction	1,487 (16.01)
Hypersensitivity	686 (7.39)
Food allergy	302 (3.25)
Asthma	255 (2.75)
Anaphylactic shock	185 (1.99)
Others	2,666 (28.70)
Unknown	3,707 (39.91)

### 3.2 AE signal detection

Table 2 shows 24 SOCs involved in AEs related to epinephrine. The five most frequent systems are general disorders and administration site conditions (*n* = 6,112, ROR: 1.75, PRR: 1.54, IC: 0.62, EBGM: 1.54), injury, poisoning and procedural complications (*n* = 4,287, ROR: 2.24, PRR: 2, IC: 1, EBGM: 2), cardiac disorders (*n* = 2,454, ROR: 4.45, PRR: 4.06, IC: 2.02, EBGM: 4.06), nervous system disorders (*n* = 1,609, ROR: 0.82, PRR: 0.83, IC: -0.26, EBGM: 0.83), and investigations (*n* = 1,285, ROR: 0.9, PRR: 0.91, IC: -0.14, EBGM: 0.91).

**Table 2:** Signal strength of adverse events of epinephrine at the system organ class level in FDA adverse event reporting system database.

Rank	System organ class	Case reports	ROR (95% CI)	PRR (95% CI)	χ^2^	IC (IC025)	EBGM (EBGM05)
1	General disorders and administration site conditions	6,112	1.75 (1.7, 1.8)	1.54 (1.51, 1.57)	1413.16	0.62 (0.58)	1.54 (1.5)
2	Injury, poisoning and procedural complications	4,287	2.24 (2.17, 2.32)	2 (1.96, 2.04)	2362.74	1 (0.95)	2 (1.94)
3	Cardiac disorders	2,454	4.45 (4.27, 4.64)	4.06 (3.9, 4.22)	5817.63	2.02 (1.96)	4.06 (3.92)
4	Nervous system disorders	1,609	0.82 (0.78, 0.86)	0.83 (0.8, 0.86)	58.41	−0.26 (−0.34)	0.83 (0.8)
5	Investigations	1,285	0.9 (0.86, 0.96)	0.91 (0.86, 0.97)	12.17	−0.14 (−0.22)	0.91 (0.87)
6	Immune system disorders	1,104	4.53 (4.27, 4.81)	4.35 (4.1, 4.61)	2879.92	2.12 (2.03)	4.35 (4.13)
7	Respiratory, thoracic and mediastinal disorders	1,076	0.99 (0.93, 1.05)	0.99 (0.93, 1.05)	0.15	−0.02 (−0.1)	0.99 (0.94)
8	Vascular disorders	901	1.87 (1.75, 2)	1.83 (1.73, 1.94)	347.92	0.87 (0.78)	1.83 (1.73)
9	Gastrointestinal disorders	604	0.29 (0.27, 0.31)	0.31 (0.29, 0.34)	1019.92	−1.69 (−1.81)	0.31 (0.29)
10	Psychiatric disorders	503	0.37 (0.34, 0.41)	0.39 (0.36, 0.42)	515.04	−1.36 (−1.49)	0.39 (0.36)
11	Skin and subcutaneous tissue disorders	480	0.38 (0.34, 0.41)	0.39 (0.35, 0.43)	483.27	−1.36 (−1.49)	0.39 (0.36)
12	Musculoskeletal and connective tissue disorders	366	0.29 (0.26, 0.32)	0.3 (0.27, 0.33)	617.48	−1.72 (−1.87)	0.3 (0.28)
13	Eye disorders	280	0.61 (0.54, 0.68)	0.61 (0.54, 0.69)	70.76	−0.71 (−0.88)	0.61 (0.55)
14	Infections and infestations	247	0.2 (0.17, 0.22)	0.21 (0.19, 0.24)	805.51	−2.29 (−2.47)	0.21 (0.18)
15	Metabolism and nutrition disorders	226	0.46 (0.4, 0.52)	0.46 (0.4, 0.53)	145.18	−1.12 (−1.3)	0.46 (0.41)
16	Renal and urinary disorders	94	0.22 (0.18, 0.27)	0.22 (0.18, 0.27)	256.81	−2.16 (−2.45)	0.22 (0.19)
17	Blood and lymphatic system disorders	63	0.16 (0.12, 0.2)	0.16 (0.12, 0.21)	278.67	−2.63 (−2.98)	0.16 (0.13)
18	Congenital, familial and genetic disorders	44	0.62 (0.46, 0.84)	0.63 (0.47, 0.85)	9.88	−0.68 (−1.1)	0.63 (0.49)
19	Pregnancy, puerperium and perinatal conditions	43	0.44 (0.33, 0.59)	0.44 (0.33, 0.59)	30.83	−1.18 (−1.61)	0.44 (0.34)
20	Ear and labyrinth disorders	30	0.3 (0.21, 0.44)	0.31 (0.22, 0.44)	47.51	−1.71 (−2.22)	0.31 (0.23)
21	Hepatobiliary disorders	23	0.11 (0.07, 0.16)	0.11 (0.07, 0.17)	167.1	−3.18 (−3.76)	0.11 (0.08)
22	Endocrine disorders	11	0.19 (0.1, 0.34)	0.19 (0.11, 0.34)	38.34	−2.4 (−3.22)	0.19 (0.12)
23	Neoplasms benign, malignant and unspecified (incl cysts and polyps)	9	0.01 (0.01, 0.03)	0.01 (0.01, 0.02)	600.78	−6.06 (−6.96)	0.01 (0.01)
24	Reproductive system and breast disorders	6	0.03 (0.01, 0.07)	0.03 (0.01, 0.07)	176.82	−4.96 (−6.03)	0.03 (0.02)

ROR, reporting odds ratio; PRR, proportional reporting ratio; IC, information component; EBGM, Empirical Bayes Geometric Mean.

A total of 264 significant PTs met all four algorithms concurrently (Supplementary Table S3), and the top 50 PTs ranked by EBGM classified by SOC are presented in Table 3. Drug ineffective (*n* = 1,867, ROR: 4.04, PRR: 3.78, IC: 1.92, EBGM: 3.78), accidental exposure to product (*n* = 952, ROR: 30.45, PRR: 29.17, IC: 4.85, EBGM: 28.83), and drug hypersensitivity (*n* = 668, ROR: 9.23, PRR: 8.98, IC: 3.16, EBGM: 8.95) were three most common PTs. Injection site ischemia (*n* = 39, ROR: 3242.28, PRR: 3236.49, IC: 10.43, EBGM: 1380.84) had the greatest signal intensity, followed by injection site pallor (*n* = 132, ROR: 2022.81, PRR: 2010.6, IC: 10.1, EBGM: 1095.88) and medical device site laceration (*n* = 9, ROR: 1547.75, PRR: 1547.11, IC: 9.88, EBGM: 942.11). Notably, myocardial stunning, systolic anterior motion of mitral valve, left ventricle outflow tract obstruction, harlequin syndrome, injection site nerve damage, and injection site movement impairment were significant AEs beyond drug labels with strong signal intensities.

**Table 3:** Top 50 signal strength of adverse events of epinephrine ranked by EBGM at the preferred term level in FDA adverse event reporting system database.

Rank	System organ class	Preferred term	Case reports	ROR (95% CI)	PRR (95% CI)	χ^2^	IC (IC025)	EBGM (EBGM05)
1	General disorders and administration site conditions	Injection site ischemia	39	3242.28 (2004.71, 5243.84)	3236.49 (1982.76, 5282.98)	53797.29	10.43 (9.87)	1380.84 (923.49)
2		Injection site pallor	132	2022.81 (1604.77, 2549.73)	2010.6 (1589.2, 2543.73)	144453.51	10.1 (9.8)	1095.88 (902.9)
3		Medical device site laceration	9	1547.75 (669.85, 3576.24)	1547.11 (666.04, 3593.72)	8464.55	9.88 (8.82)	942.11 (467.48)
4		Injection site anesthesia	46	400.5 (293.06, 547.33)	399.66 (292.08, 546.87)	15687.28	8.42 (7.98)	342.88 (264.02)
5		Application site pallor	4	343.87 (120.6, 980.44)	343.8 (121.66, 971.52)	1196.32	8.23 (6.88)	300.95 (125.24)
6		Injection site laceration	68	315.09 (244.64, 405.82)	314.11 (243.46, 405.27)	18773.52	8.12 (7.76)	277.96 (224.92)
7		Injection site coldness	64	287.13 (221.5, 372.2)	286.29 (221.9, 369.37)	16260.62	8 (7.63)	255.96 (206)
8		Injection site movement impairment	8	183.43 (89.37, 376.47)	183.36 (88.79, 378.66)	1348.22	7.41 (6.43)	170.45 (93.39)
9		Injection site hypoesthesia	72	153.71 (121.08, 195.13)	153.21 (121.1, 193.84)	10235.98	7.17 (6.83)	144.1 (118.02)
10		Injection site injury	109	64.05 (52.93, 77.5)	63.73 (52.39, 77.53)	6557.41	5.96 (5.68)	62.11 (52.95)
11		Injection site nerve damage	4	45.2 (16.81, 121.56)	45.19 (16.96, 120.41)	169.68	5.47 (4.19)	44.38 (19.4)
12		Injection site paresthesia	23	45.05 (29.82, 68.06)	45 (29.82, 67.92)	971.41	5.47 (4.88)	44.19 (31.29)
13		Application site hematoma	3	41.5 (13.25, 129.94)	41.49 (13.31, 129.32)	116.54	5.35 (3.92)	40.81 (15.7)
14	Investigations	End-tidal CO2 decreased	3	185.15 (57.21, 599.18)	185.12 (57.11, 600.04)	510.15	7.43 (5.95)	171.97 (64.37)
15		Electrocardiogram ST-T segment depression	3	131.29 (41.07, 419.7)	131.27 (41.3, 417.24)	367.77	6.96 (5.5)	124.53 (47.09)
16		Epinephrine increased	4	113.27 (41.55, 308.8)	113.25 (41.68, 307.72)	425.05	6.76 (5.46)	108.21 (46.75)
17		Capillary nail refill test abnormal	4	108.18 (39.72, 294.62)	108.16 (39.81, 293.89)	406.42	6.69 (5.4)	103.55 (44.78)
18		Cardiac index decreased	4	103.53 (38.05, 281.69)	103.51 (38.09, 281.26)	389.34	6.63 (5.34)	99.28 (42.97)
19		ECG signs of myocardial ischemia	7	81.8 (38.51, 173.77)	81.78 (38.83, 172.23)	540.18	6.31 (5.29)	79.12 (42.12)
20		Mean arterial pressure decreased	6	66.56 (29.57, 149.81)	66.54 (29.79, 148.62)	376.93	6.02 (4.93)	64.78 (32.86)
21		Radial pulse abnormal	3	64.47 (20.48, 202.95)	64.46 (20.68, 200.91)	182.55	5.97 (4.53)	62.81 (24.06)
22		Electrocardiogram ST segment depression	44	51.86 (38.46, 69.93)	51.76 (38.58, 69.45)	2144.06	5.66 (5.24)	50.69 (39.47)
23		Pulse pressure increased	4	49.63 (18.44, 133.58)	49.62 (18.26, 134.83)	186.72	5.6 (4.32)	48.64 (21.24)
24		Troponin T increased	18	38.81 (24.36, 61.84)	38.78 (24.23, 62.07)	652.04	5.25 (4.6)	38.18 (25.86)
25		Electrocardiogram ST segment elevation	46	36.28 (27.1, 48.55)	36.2 (26.98, 48.57)	1551.3	5.16 (4.74)	35.68 (27.96)
26	Cardiac disorders	Systolic anterior motion of mitral valve	13	549.2 (300.64, 1003.28)	548.88 (298.95, 1007.75)	5789.13	8.8 (7.98)	447.13 (270.07)
27		Myocardial stunning	10	248.22 (129.44, 476)	248.11 (129.94, 473.75)	2231.09	7.81 (6.92)	225.01 (130.5)
28		Stress cardiomyopathy	300	156.84 (139.45, 176.39)	154.7 (137.54, 174.01)	43049.14	7.18 (7.02)	145.42 (131.8)
29		Kounis syndrome	66	85.84 (67.13, 109.76)	85.58 (66.33, 110.42)	5327.82	6.37 (6.02)	82.68 (67.3)
30		Arteriospasm coronary	88	63.26 (51.17, 78.21)	63.01 (50.79, 78.17)	5233.76	5.94 (5.64)	61.43 (51.44)
31		Hyperdynamic left ventricle	4	45.85 (17.05, 123.31)	45.84 (17.2, 122.14)	172.17	5.49 (4.21)	45 (19.67)
32		Myocardial necrosis	4	44.37 (16.5, 119.3)	44.36 (16.65, 118.2)	166.47	5.45 (4.17)	43.58 (19.05)
33	Injury, poisoning and procedural complications	Product design confusion	4	131.89 (48.2, 360.89)	131.87 (48.53, 358.32)	492.52	6.97 (5.66)	125.07 (53.87)
34		Wrong product stored	9	82.08 (42.23, 159.52)	82.04 (42.13, 159.75)	696.76	6.31 (5.4)	79.37 (45.52)
35		Accidental exposure to product by child	190	77.91 (67.39, 90.07)	77.24 (67.34, 88.6)	13854.97	6.23 (6.02)	74.87 (66.31)
36		Cataract operation complication	12	62.82 (35.41, 111.44)	62.78 (35.56, 110.83)	711.03	5.94 (5.14)	61.21 (37.89)
37		Product appearance confusion	16	49.34 (30.07, 80.95)	49.3 (30.2, 80.47)	741.99	5.59 (4.9)	48.33 (31.94)
38		Product packaging confusion	39	42.76 (31.14, 58.7)	42.68 (31.19, 58.4)	1559.92	5.39 (4.94)	41.96 (32.18)
39	Vascular disorders	Vasoconstriction	33	112.02 (79, 158.86)	111.86 (78.61, 159.18)	3464.6	6.74 (6.24)	106.93 (79.83)
40		Diastolic hypertension	4	83 (30.63, 224.91)	82.99 (30.54, 225.5)	313.2	6.33 (5.04)	80.25 (34.85)
41		Dry gangrene	11	45.35 (24.97, 82.36)	45.33 (25.18, 81.61)	468.06	5.48 (4.65)	44.51 (27.02)
42		Vasospasm	14	41.27 (24.33, 70)	41.24 (24.29, 70.01)	540.44	5.34 (4.61)	40.56 (26.07)
43		Systolic hypertension	6	35.75 (15.96, 80.06)	35.74 (16, 79.83)	199.66	5.14 (4.06)	35.23 (17.95)
44	Gastrointestinal disorders	Intestinal hematoma	4	64.19 (23.78, 173.28)	64.18 (23.62, 174.39)	242.31	5.97 (4.68)	62.54 (27.24)
45		Gastrointestinal ischemia	5	46.83 (19.33, 113.49)	46.82 (19.38, 113.1)	219.94	5.52 (4.35)	45.95 (21.91)
46	Pregnancy, puerperium and perinatal conditions	Uterine hypertonus	4	37.46 (13.95, 100.59)	37.46 (14.06, 99.81)	139.76	5.21 (3.93)	36.9 (16.15)
47	Nervous system disorders	Harlequin syndrome	3	124.5 (39.01, 397.37)	124.48 (39.16, 395.66)	349.39	6.89 (5.43)	118.41 (44.84)
48	Musculoskeletal and connective tissue disorders	Chondrolysis	10	52.46 (28.03, 98.16)	52.43 (28, 98.17)	493.76	5.68 (4.82)	51.34 (30.39)
49	Infections and infestations	Gas gangrene	10	78.43 (41.77, 147.25)	78.39 (41.87, 146.77)	739.95	6.25 (5.38)	75.95 (44.83)
50	Congenital, familial and genetic disorders	Left ventricle outflow tract obstruction	22	179.66 (116.47, 277.11)	179.48 (116.61, 276.24)	3633.65	7.38 (6.77)	167.09 (116.27)

ROR, reporting odds ratio; PRR, proportional reporting ratio; IC, information component; EBGM, Empirical Bayes Geometric Mean.

### 3.3 Time to onset analysis

**Figure 2:**
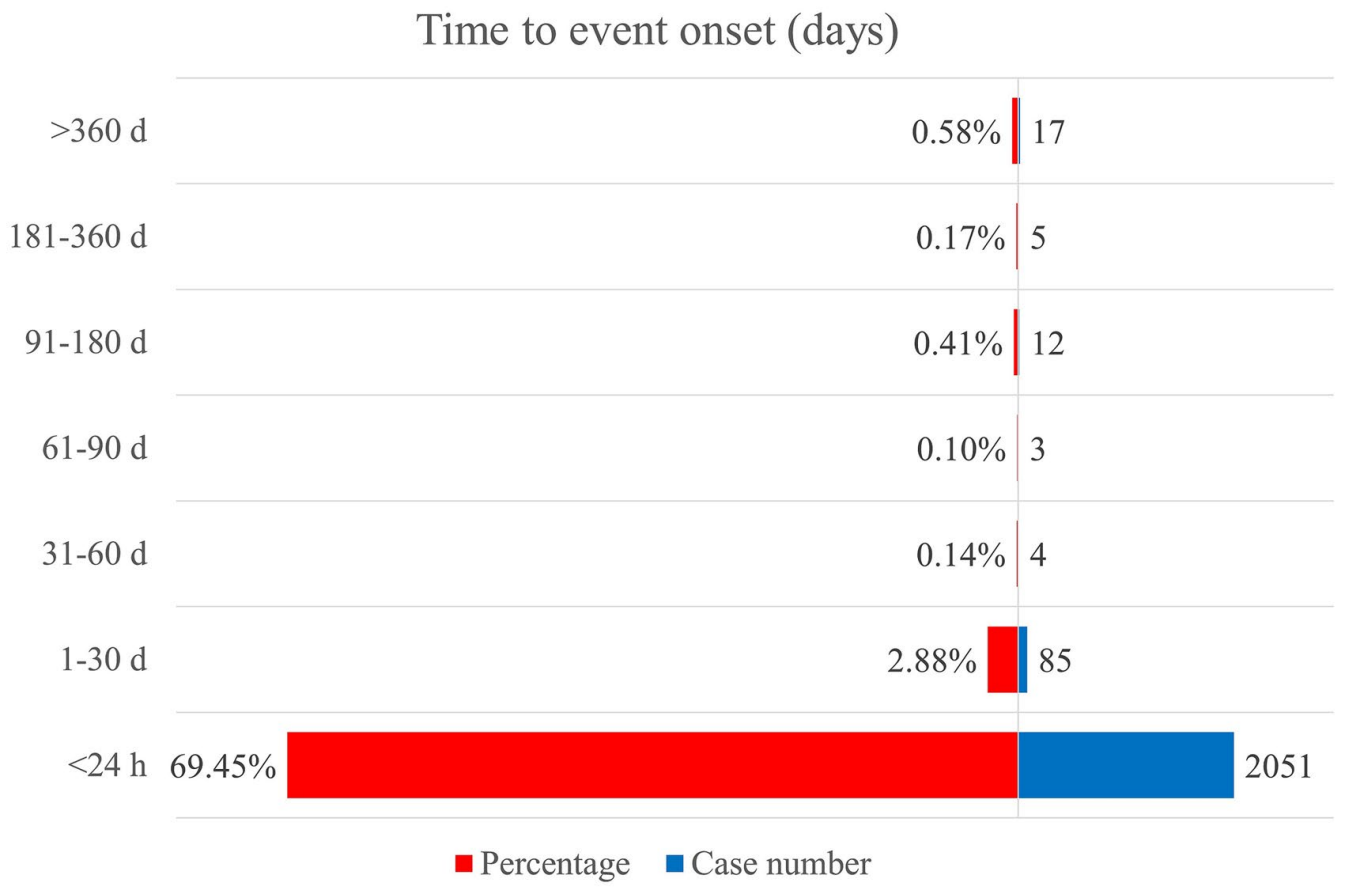
Time to onset of adverse events related to epinephrine.

Solid onset times were provided by 2,177 AE reports related to epinephrine with the median time to onset of 0 day (interquartile range [IQR] 0–0 day). Most of AEs occurred within 24 h after epinephrine administration (*n* = 2,051, 69.45%). Details are shown in Figure 2.

### 3.4 Subgroup analysis

The PT with highest signal strength was injection site ischaemia in subgroup of intramuscular (IM) and SC epinephrine, and systolic anterior motion of mitral valve in subgroup of IV route. Drug ineffective, stress cardiomyopathy, and accidental exposure to product were the most frequent AE in IM, IV, and SC route of administration, respectively (Supplementary Tables S4–S6).

## 4 Discussion

To our knowledge, this is the first investigation to assess the relationship between epinephrine usage and associated AEs under real-world conditions, utilizing data from the FAERS pharmacovigilance system. This study examined cases from the first quarter of 2004 to the fourth quarter of 2024, revealing 24 implicated SOCs and identifying 264 significant PTs, while also uncovering several previously unrecognized risks. These findings provide important supplementary insights to further support clinical decision-making processes.

The most common route of administration and indication among epinephrine-associated AE reports were IM (15.17%) and anaphylactic reaction (16.01%) respectively. This may be because epinephrine is primarily indicated for anaphylaxis, and IM administration is widely recognized as the first-line therapy (1). The proportion of female was far higher than that of male, as increased risk of anaphylaxis in women has been proved by multiple epidemiologic studies (9). Most of the AE reports originated from healthcare providers, suggesting that the adverse event data in this investigation is both reliable and thorough.

In previous studies, two reviews indicated that the mild transient AEs of epinephrine for anaphylaxis treatment included palpitations, headache, pallor, tremor, anxiety, and dizziness, while pulmonary edema, hypertension, ventricular arrhythmias, and myocardial infarction were severe AEs (8, 10). Four retrospective studies revealed that epinephrine-associated AEs comprised arrhythmia, ischemia, angina, incidental elevated troponin, hypotension, hypertension, chest tightness, coronary vasospasm, myocardial infarction, palpitations, chest pain, ECG abnormalities, tremors, anxiety, paleness, cold, headache, and hotness (11–14). Two studies conducted by Pouessel et al. in France demonstrated that tachycardia, hypertension, chest tightness, chest pain, lower limb vasospasm, induration at the injection site, pain, pallor, coldness, hematoma, hypoesthesia-paresthesia, ischemia, dizziness, tremors, and vomiting were AE related to epinephrine auto-injectors (EAIs) (15, 16). Several case reports showed that the epinephrine administration may induce multifocal atrial tachycardia, intracerebral hemorrhage, myocardial infarction, digital ischemia, and transient myocardial ischemia (17–22).

Consistent with these findings, we discovered that the general disorders and administration site conditions was the most prevalent SOC in this study, which included pallor, ischemia, laceration, coldness, paresthesia, and hematoma in injection site. There were 300 cases of stress cardiomyopathy, 33 cases of vasoconstriction, 14 cases of vasospasm, and 10 cases of hypertension in SOCs of cardiac disorders and vascular disorders. The signal intensity of ECG signs of myocardial ischemia in the SOC of investigations was ROR 81.8, PRR 81.78, IC 6.31, and EBGM 79.12. Moreover, among the SOC of infections and infestations, we identified 10 cases of gas gangrene. These AE signals were in accordance with drug instructions, validating the reliability, credibility, and robustness of our research.

It is noteworthy that we identify several novel AE signals, including myocardial stunning, systolic anterior motion of mitral valve, left ventricle outflow tract obstruction, harlequin syndrome, injection site nerve damage, and injection site movement impairment.

The signal intensity of myocardial stunning is ROR 248.22, PRR 248.11, IC 7.81, and EBGM 225.01. A case report showed that a middle-aged female developed myocardial stunning after accidental epinephrine injection (23). Morel et al. emphasized that epinephrine may lead to transient left ventricular dysfunction syndrome through myocardial stunning (24). While the mechanism remains unclear, the possible explanations are as follows: Excessive concentrations of epinephrine are thought to trigger coronary vasospasms and microvascular dysfunction through alpha-adrenergic receptor activation, alongside elevations in blood pressure and ventricular afterload, ultimately leading to myocardial stunning due to ischemia. Additionally, epinephrine can engage beta-adrenergic receptors (βAR), particularly β1AR-Gs, thereby enhancing oxidative stress and inducing temporary hypercontractility. At even higher concentrations, epinephrine stimulates β2AR-Gi receptors, which exert a negative inotropic effect, contributing to direct myocardial stunning. This phenomenon serves a cardioprotective role by minimizing myocyte necrosis and facilitating myocardial recovery through the activation of anti-apoptotic mechanisms and the phosphoinositide 3-kinase/protein kinase B signaling pathway (25).

Both left ventricle outflow tract (LVOT) obstruction and systolic anterior motion (SAM) of mitral valve possess strong signal strength. The pathognomonic features of hypertrophic cardiomyopathy (HCM) consist of asymmetric left ventricular hypertrophy, LVOT obstruction and SAM of the mitral valve (26). It was reported that HCM was caused by catecholamine-producing tumor in a 37-year-old woman (27). These AEs may result from the following reason: Epinephrine increases myocardial contractility and accelerates left ventricular systole, leading to a higher LVOT pressure gradient. This effect can draw the anterior mitral valve leaflet into the LVOT, causing SAM of mitral valve, and further leading to LVOT obstruction.

Harlequin syndrome (HS) is a rare AE highly associated with epinephrine in this study (*n* = 3, ROR: 124.5, PRR: 124.48, IC: 6.89, EBGM: 118.41). It is an autonomic syndrome with the symptoms of sudden occurrence of unilateral flushing and sweating on the face and/or arm (28). The primary factor contributing to HS is the interruption of sympathetic pathways in affected side (29). Epinephrine administration may exacerbate the manifestations of HS by activating the sympathetic nervous system. Due to unilateral sympathetic dysfunction, the affected side has impaired sweating and vasomotor control, while the intact side remains responsive, leading to exaggerated flushing and sweating on the contralateral face.

Both injection site nerve damage and injection site movement impairment show high signal intensity. Vasoconstriction and mechanical injury caused by epinephrine application may further lead to nerve damage at the injection site, potentially resulting in motor dysfunction at the affected area (30).

Our findings showed that the median time to onset was 0 day and the majority of AEs emerged at epinephrine use for less than 24 h (69.45%). It provides essential references for the setting of follow-up period in future researches, and emphasizes that patients receiving epinephrine should be immediately and closely monitored by health professionals to promptly detect any abnormality.

The safety of different routes of administration varies, and it is generally believed that IM epinephrine is safer than IV administration. IV epinephrine is associated with higher incidence and severity of adverse effect (8). Campbell et al. conducted an observational cohort study of 573 patients and demonstrated that the risk of cardiovascular adverse event is greater in IV bolus epinephrine than IM route in anaphylaxis management (11). Conversely, a recent retrospective study showed that continuous IV infusion of epinephrine provided better safety outcome compared to IM injection during anaphylaxis treatment in 142 Japanese (31). We, respectively, present the safety profiles of three most common routes of epinephrine administration in collected AE reports, which allows physicians to comprehensively monitor the conditions of patients and further ensure safety after epinephrine application.

Notably, we determined 190 cases of accidental exposure to product by child, which is consistent of previous studies. Pouessel et al. reported 10 pediatric cases with accidental injection of EAIs based on French pharmacovigilance database (15). Furthermore, 57% cases of unintentional injections from EAIs were less than 18 years old according to American Association of Poison Control Centers database from 1994 to 2007 (32). It highlights the importance of enhancing education for parents and caregivers to properly store EAIs. Besides, Manufacturers should improve the safety-locking mechanisms of EAIs to minimize accidental activation by children.

Strong signal intensities were found among injection site ischemia, injection site pallor, injection site nerve damage, injection site movement impairment, and medical device site laceration, which is consistent with previous studies (33). To address this issue, several needle-free devices for nasal or sublingual route were under development and assessment, which could eliminate the injury associated with needle, adaptability of needle-length, and needle-phobia, thereby promoting the easy and convenient epinephrine administration. These products had demonstrated comparable pharmacokinetic and pharmacodynamic outcome compared to standard treatment of EAI and manual IM injection (34). Based on these evidences, FDA has already approved intranasal epinephrine device at 09 August 2024 (35). It underscores the urgent need for developing and upgrading the route of administration of epinephrine to further restrain the AEs and ameliorate the effectiveness.

This research is subject to several limitations. Firstly, AEs are voluntarily reported to the FAERS database, resulting in submissions from reporters with varying medical backgrounds. Consequently, some of the information provided may lack completeness, potentially leading to biased results. Secondly, the occurrence of AEs could be influenced by numerous confounders, including co-administered medications and underlying comorbid conditions. Thirdly, our investigation employed disproportionality analysis exclusively to evaluate signal strength, thereby establishing only an association between epinephrine administration and reported AEs, without confirming a definitive causal relationship. Fourth, this research fails to include the data of AEs where epinephrine was administered as part of a combination medicinal product. Lastly, since a substantial proportion of the reports were sourced from the United States, generalizability to other populations may be limited. Despite these constraints, this study contributes valuable evidence to clinical practice, enhancing the existing knowledge and improving understanding regarding the safety profile of epinephrine.

## 5 Conclusion

In conclusion, this research systematically analyzed data from the FAERS database, revealing both the range of adverse event signals associated with epinephrine and their onset timing. Our findings corroborate previously recognized adverse reactions documented on the drug’s labeling information, and notably, we identified several previously unreported adverse event signals, such as myocardial stunning, systolic anterior motion of mitral valve, left ventricle outflow tract obstruction, harlequin syndrome, injection site nerve damage, and injection site movement impairment. These newly discovered signals provide valuable guidance and a solid foundation for future in-depth research.

## Data Availability

Publicly available datasets were analyzed in this study. This data can be found here: https://www.fda.gov/drugs/drug-approvals-and-databases/fda-adverse-event-reporting-system-faers-database.
