# Supplementary material for: Post-marketing safety assessment of epinephrine: an analysis of the US FDA adverse event reporting system
Source: Front Med (Lausanne). 2025 Dec 18;12:1727631. doi: 10.3389/fmed.2025.1727631 (PMC12756475; doi:10.3389/fmed.2025.1727631)
Supplement: Supplementary file 1 [file Data_Sheet_1.docx]

Supplementary materials

Table S1 Four grid table.

|  | Epinephrine-related adverse events | Non-epinephrine-related adverse events | Total |
| --- | --- | --- | --- |
| Epinephrine | a | b | a + b |
| Non-epinephrine | c | d | c + d |
| Total | a + c | b + d | N = a + b + c + d |

Table S2 Four major algorithms used to explore the potential association between epinephrine and adverse events.

| Method | Formula | ﻿Threshold |
| --- | --- | --- |
| ROR | $ROR=\frac{a / c}{b / d}$ | a ≥ 3, ﻿95% CI (lower limit) > 1 |
|  | $SE(lnROR)=\sqrt{\frac{1}{a}+\frac{1}{b}+\frac{1}{c}+\frac{1}{d}}$ |  |
|  | $95\%CI= e^{\ln\left( ROR \right)\pm1.96se}$ |  |
| PRR | $PRR=\frac{a / (a+b)}{c / (c+d)}$ | a ≥ 3, 95% CI (lower limit) > 1 |
|  | $SE(lnPRR)=\sqrt{\frac{1}{a}-\frac{1}{a+b}+\frac{1}{c}-\frac{1}{c+d}}$ |  |
|  | $95\%CI= e^{\ln\left( PRR \right)\pm1.96se}$ |  |
| BCPNN | $IC=\log_{2}\frac{p(x, y)}{p\left( x \right)p(y)}= {log}_{2}\frac{a(a+b+c+d)}{(a+b)(a+c)}$ | IC025 > 0 |
|  | $E(IC)=\log_{2}\frac{(a+\gamma11)(a+b+c+d+\alpha)(a+b+c+d+\beta)}{\left( a+b+c+d+\gamma\right)(a+b+\alpha1)(a+c+\beta1)}$ |  |
|  | $V\left( \mathrm{IC} \right)=\frac{1}{{(ln2)}^{2}}[\frac{\left( a+b+c+d \right)-a+\gamma-\gamma11}{\left( a+\gamma11 \right)\left( 1+a+b+c+d+\gamma\right)}+\frac{\left( a+b+c+d \right)-\left( a+b \right)+a-\alpha1}{\left( a+b+\alpha1 \right)\left( 1+a+b+c+d+\alpha\right)}+\frac{\left( a+b+c+d+\alpha\right)-\left( a+c \right)+\beta-\beta1}{\left( a+b+\beta1 \right)\left( 1+a+b+c+d+\beta\right)}]$ |  |
|  | $\gamma=\gamma11\frac{(a+b+c+d+\alpha)(a+b+c+d+\beta)}{\left( a+b+\alpha1 \right)(a+c+\beta1)}$ |  |
|  | $IC-2SD=E\left( \mathrm{IC} \right)-2 \sqrt{V(IC)}$ |  |
| EBGM | $EBGM=\frac{a(a+b+c+d)}{\left( a+c \right)(a+b)}$ | EBGM05 > 2 |
|  | $SE(lnEBGM)=\sqrt{\frac{1}{a}+\frac{1}{b}+\frac{1}{c}+\frac{1}{d}}$ |  |
|  | $95\%CI= e^{\ln\left( EBGM \right)\pm1.96se}$ |  |

Abbreviations: ROR, reporting odds ratio; PRR, proportional reporting ratio; BCPNN, Bayesian Confidence Propagation Neural Network; EBGM, Empirical Bayes Geometric Mean.

Table S3 Signal strength of adverse events of epinephrine ranked by EBGM at the preferred term level in FDA adverse event reporting system database.

| Rank | System organ class | Preferred term | Case reports | ROR (95% CI) | PRR (95% CI) | χ^2^ | IC (IC025) | EBGM (EBGM05) |
| --- | --- | --- | --- | --- | --- | --- | --- | --- |
| 1 | Injury, poisoning and procedural complications | Product design confusion | 4 | 131.89 (48.2, 360.89) | 131.87 (48.53, 358.32) | 492.52 | 6.97 (5.66) | 125.07 (53.87) |
| 2 |  | Wrong product stored | 9 | 82.08 (42.23, 159.52) | 82.04 (42.13, 159.75) | 696.76 | 6.31 (5.4) | 79.37 (45.52) |
| 3 |  | Accidental exposure to product by child | 190 | 77.91 (67.39, 90.07) | 77.24 (67.34, 88.6) | 13854.97 | 6.23 (6.02) | 74.87 (66.31) |
| 4 |  | Cataract operation complication | 12 | 62.82 (35.41, 111.44) | 62.78 (35.56, 110.83) | 711.03 | 5.94 (5.14) | 61.21 (37.89) |
| 5 |  | Product appearance confusion | 16 | 49.34 (30.07, 80.95) | 49.3 (30.2, 80.47) | 741.99 | 5.59 (4.9) | 48.33 (31.94) |
| 6 |  | Product packaging confusion | 39 | 42.76 (31.14, 58.7) | 42.68 (31.19, 58.4) | 1559.92 | 5.39 (4.94) | 41.96 (32.18) |
| 7 |  | Maternal exposure during delivery | 10 | 34.2 (18.32, 63.85) | 34.18 (18.26, 64) | 317.63 | 5.08 (4.22) | 33.72 (20) |
| 8 |  | Wrong drug | 4 | 33.78 (12.59, 90.64) | 33.78 (12.68, 90.01) | 125.47 | 5.06 (3.78) | 33.32 (14.59) |
| 9 |  | Nail injury | 7 | 33.77 (16.01, 71.21) | 33.76 (16.03, 71.1) | 219.45 | 5.06 (4.05) | 33.31 (17.84) |
| 10 |  | Local anaesthetic systemic toxicity | 8 | 33.61 (16.73, 67.55) | 33.6 (16.59, 68.04) | 249.56 | 5.05 (4.1) | 33.15 (18.49) |
| 11 |  | Toxic anterior segment syndrome | 13 | 32.57 (18.84, 56.32) | 32.56 (18.81, 56.37) | 392.32 | 5.01 (4.24) | 32.13 (20.33) |
| 12 |  | Exposure via eye contact | 4 | 30.57 (11.4, 81.95) | 30.56 (11.47, 81.43) | 112.94 | 4.92 (3.64) | 30.19 (13.23) |
| 13 |  | Accidental exposure to product | 952 | 30.45 (28.53, 32.51) | 29.17 (27.5, 30.94) | 25628.48 | 4.85 (4.76) | 28.83 (27.3) |
| 14 |  | Product selection error | 12 | 29.07 (16.45, 51.37) | 29.05 (16.45, 51.29) | 321.19 | 4.84 (4.05) | 28.72 (17.83) |
| 15 |  | Expired device used | 31 | 28.2 (19.79, 40.19) | 28.16 (19.79, 40.07) | 802.82 | 4.8 (4.3) | 27.85 (20.7) |
| 16 |  | Expired product administered | 346 | 28.57 (25.67, 31.79) | 28.13 (25.5, 31.03) | 8953.63 | 4.8 (4.64) | 27.82 (25.44) |
| 17 |  | Device use issue | 151 | 26.29 (22.38, 30.88) | 26.11 (22.32, 30.54) | 3608.51 | 4.69 (4.46) | 25.84 (22.59) |
| 18 |  | Injury corneal | 5 | 26.11 (10.81, 63.03) | 26.1 (10.8, 63.05) | 119.41 | 4.69 (3.53) | 25.83 (12.36) |
| 19 |  | Circumstance or information capable of leading to device use error | 10 | 21.16 (11.35, 39.43) | 21.15 (11.3, 39.6) | 190.28 | 4.39 (3.53) | 20.97 (12.46) |
| 20 |  | Product label confusion | 40 | 19.85 (14.54, 27.1) | 19.82 (14.48, 27.12) | 708.88 | 4.3 (3.85) | 19.66 (15.15) |
| 21 |  | Vasoplegia syndrome | 10 | 18.74 (10.06, 34.91) | 18.73 (10, 35.07) | 166.53 | 4.22 (3.36) | 18.59 (11.04) |
| 22 |  | Wrong device used | 4 | 17.54 (6.56, 46.9) | 17.53 (6.58, 46.71) | 61.92 | 4.12 (2.85) | 17.41 (7.65) |
| 23 |  | Product name confusion | 8 | 17.18 (8.57, 34.44) | 17.17 (8.65, 34.1) | 121 | 4.09 (3.15) | 17.06 (9.53) |
| 24 |  | Foetal exposure during delivery | 4 | 16.13 (6.03, 43.12) | 16.12 (6.05, 42.95) | 56.37 | 4 (2.73) | 16.02 (7.04) |
| 25 |  | Product confusion | 4 | 15.48 (5.79, 41.38) | 15.48 (5.81, 41.25) | 53.82 | 3.94 (2.67) | 15.38 (6.76) |
| 26 |  | Intercepted product selection error | 4 | 14.46 (5.41, 38.64) | 14.45 (5.42, 38.5) | 49.8 | 3.85 (2.58) | 14.37 (6.31) |
| 27 |  | Incorrect route of product administration | 76 | 13.36 (10.66, 16.75) | 13.32 (10.74, 16.52) | 861.59 | 3.73 (3.4) | 13.25 (10.97) |
| 28 |  | Skeletal injury | 52 | 12.41 (9.45, 16.31) | 12.39 (9.42, 16.3) | 541.57 | 3.62 (3.23) | 12.33 (9.81) |
| 29 |  | Wrong product administered | 27 | 11.93 (8.17, 17.41) | 11.91 (8.21, 17.28) | 268.58 | 3.57 (3.03) | 11.86 (8.64) |
| 30 |  | Iatrogenic injury | 6 | 11.65 (5.22, 25.98) | 11.64 (5.21, 26) | 58.11 | 3.54 (2.46) | 11.59 (5.93) |
| 31 |  | Intercepted product dispensing error | 4 | 9.99 (3.74, 26.67) | 9.99 (3.75, 26.62) | 32.21 | 3.31 (2.05) | 9.95 (4.37) |
| 32 |  | Peripheral nerve injury | 3 | 9.63 (3.1, 29.92) | 9.63 (3.09, 30.01) | 23.1 | 3.26 (1.84) | 9.59 (3.71) |
| 33 |  | Prescription drug used without a prescription | 17 | 8.27 (5.14, 13.32) | 8.27 (5.17, 13.24) | 108.23 | 3.04 (2.38) | 8.24 (5.53) |
| 34 |  | Procedural haemorrhage | 11 | 8.13 (4.5, 14.7) | 8.13 (4.52, 14.64) | 68.56 | 3.02 (2.2) | 8.11 (4.94) |
| 35 |  | Arthropod sting | 5 | 8.02 (3.33, 19.29) | 8.02 (3.32, 19.37) | 30.61 | 3 (1.84) | 7.99 (3.83) |
| 36 |  | Accidental overdose | 95 | 7.29 (5.96, 8.92) | 7.27 (5.98, 8.84) | 512.08 | 2.86 (2.57) | 7.25 (6.12) |
| 37 |  | Off label use of device | 3 | 6.8 (2.19, 21.12) | 6.8 (2.18, 21.19) | 14.79 | 2.76 (1.35) | 6.78 (2.63) |
| 38 |  | Product preparation issue | 12 | 6.55 (3.71, 11.54) | 6.54 (3.7, 11.55) | 56.2 | 2.71 (1.92) | 6.53 (4.06) |
| 39 |  | Medication error | 116 | 5.64 (4.7, 6.77) | 5.61 (4.7, 6.69) | 439.19 | 2.49 (2.22) | 5.6 (4.81) |
| 40 |  | Brain herniation | 6 | 5.35 (2.4, 11.92) | 5.35 (2.4, 11.95) | 21.16 | 2.42 (1.35) | 5.34 (2.73) |
| 41 |  | Anaesthetic complication | 5 | 4.89 (2.03, 11.76) | 4.89 (2.02, 11.81) | 15.44 | 2.29 (1.13) | 4.88 (2.34) |
| 42 |  | Foreign body | 8 | 4.87 (2.43, 9.74) | 4.87 (2.45, 9.67) | 24.52 | 2.28 (1.34) | 4.86 (2.72) |
| 43 |  | Circumstance or information capable of leading to medication error | 60 | 4.86 (3.77, 6.26) | 4.85 (3.76, 6.26) | 183.04 | 2.28 (1.91) | 4.84 (3.92) |
| 44 |  | Wrong dose | 4 | 4.66 (1.75, 12.42) | 4.66 (1.75, 12.42) | 11.47 | 2.22 (0.95) | 4.65 (2.05) |
| 45 |  | Incorrect dose administered by device | 34 | 4.24 (3.03, 5.94) | 4.23 (3.03, 5.9) | 83.87 | 2.08 (1.6) | 4.23 (3.19) |
| 46 |  | Incorrect drug administration rate | 6 | 3.99 (1.79, 8.88) | 3.99 (1.79, 8.91) | 13.4 | 1.99 (0.92) | 3.98 (2.04) |
| 47 |  | Product storage error | 94 | 3.24 (2.64, 3.96) | 3.23 (2.66, 3.93) | 144.33 | 1.69 (1.4) | 3.22 (2.72) |
| 48 |  | Scar | 21 | 3.22 (2.1, 4.94) | 3.22 (2.09, 4.96) | 32.03 | 1.68 (1.08) | 3.21 (2.25) |
| 49 |  | Drug dose omission by device | 70 | 2.88 (2.28, 3.64) | 2.87 (2.27, 3.63) | 85.54 | 1.52 (1.19) | 2.87 (2.36) |
| 50 | Investigations | End-tidal co2 decreased | 3 | 185.15 (57.21, 599.18) | 185.12 (57.11, 600.04) | 510.15 | 7.43 (5.95) | 171.97 (64.37) |
| 51 |  | Electrocardiogram st-t segment depression | 3 | 131.29 (41.07, 419.7) | 131.27 (41.3, 417.24) | 367.77 | 6.96 (5.5) | 124.53 (47.09) |
| 52 |  | Epinephrine increased | 4 | 113.27 (41.55, 308.8) | 113.25 (41.68, 307.72) | 425.05 | 6.76 (5.46) | 108.21 (46.75) |
| 53 |  | Capillary nail refill test abnormal | 4 | 108.18 (39.72, 294.62) | 108.16 (39.81, 293.89) | 406.42 | 6.69 (5.4) | 103.55 (44.78) |
| 54 |  | Cardiac index decreased | 4 | 103.53 (38.05, 281.69) | 103.51 (38.09, 281.26) | 389.34 | 6.63 (5.34) | 99.28 (42.97) |
| 55 |  | Ecg signs of myocardial ischaemia | 7 | 81.8 (38.51, 173.77) | 81.78 (38.83, 172.23) | 540.18 | 6.31 (5.29) | 79.12 (42.12) |
| 56 |  | Mean arterial pressure decreased | 6 | 66.56 (29.57, 149.81) | 66.54 (29.79, 148.62) | 376.93 | 6.02 (4.93) | 64.78 (32.86) |
| 57 |  | Radial pulse abnormal | 3 | 64.47 (20.48, 202.95) | 64.46 (20.68, 200.91) | 182.55 | 5.97 (4.53) | 62.81 (24.06) |
| 58 |  | Electrocardiogram st segment depression | 44 | 51.86 (38.46, 69.93) | 51.76 (38.58, 69.45) | 2144.06 | 5.66 (5.24) | 50.69 (39.47) |
| 59 |  | Pulse pressure increased | 4 | 49.63 (18.44, 133.58) | 49.62 (18.26, 134.83) | 186.72 | 5.6 (4.32) | 48.64 (21.24) |
| 60 |  | Troponin t increased | 18 | 38.81 (24.36, 61.84) | 38.78 (24.23, 62.07) | 652.04 | 5.25 (4.6) | 38.18 (25.86) |
| 61 |  | Electrocardiogram st segment elevation | 46 | 36.28 (27.1, 48.55) | 36.2 (26.98, 48.57) | 1551.3 | 5.16 (4.74) | 35.68 (27.96) |
| 62 |  | Corneal reflex decreased | 5 | 35.5 (14.68, 85.86) | 35.5 (14.7, 85.76) | 165.19 | 5.13 (3.96) | 34.99 (16.71) |
| 63 |  | Troponin i increased | 15 | 24.41 (14.67, 40.6) | 24.39 (14.65, 40.6) | 333.12 | 4.59 (3.88) | 24.16 (15.78) |
| 64 |  | Electrocardiogram t wave inversion | 18 | 20.78 (13.07, 33.06) | 20.77 (12.98, 33.25) | 335.78 | 4.36 (3.71) | 20.6 (13.97) |
| 65 |  | Cardiac output decreased | 5 | 15.29 (6.35, 36.85) | 15.29 (6.33, 36.94) | 66.36 | 3.93 (2.77) | 15.2 (7.28) |
| 66 |  | Electrocardiogram st-t change | 4 | 13.97 (5.23, 37.34) | 13.97 (5.24, 37.22) | 47.9 | 3.8 (2.53) | 13.9 (6.11) |
| 67 |  | Blood creatine phosphokinase mb increased | 7 | 13.28 (6.32, 27.92) | 13.28 (6.31, 27.97) | 79.02 | 3.72 (2.72) | 13.21 (7.09) |
| 68 |  | Electrocardiogram st segment abnormal | 4 | 13.05 (4.88, 34.86) | 13.04 (4.89, 34.74) | 44.24 | 3.7 (2.43) | 12.98 (5.7) |
| 69 |  | Tryptase increased | 3 | 12.8 (4.12, 39.82) | 12.8 (4.11, 39.89) | 32.47 | 3.67 (2.25) | 12.74 (4.93) |
| 70 |  | Anion gap | 3 | 11.67 (3.75, 36.27) | 11.66 (3.74, 36.34) | 29.11 | 3.54 (2.12) | 11.61 (4.49) |
| 71 |  | Electrocardiogram change | 4 | 10.94 (4.1, 29.22) | 10.94 (4.11, 29.15) | 35.96 | 3.45 (2.18) | 10.89 (4.79) |
| 72 |  | Pco2 increased | 3 | 10.78 (3.47, 33.5) | 10.78 (3.46, 33.6) | 26.49 | 3.42 (2.01) | 10.73 (4.15) |
| 73 |  | Troponin increased | 28 | 10.62 (7.33, 15.4) | 10.61 (7.31, 15.4) | 242.68 | 3.4 (2.88) | 10.57 (7.74) |
| 74 |  | Blood lactic acid increased | 20 | 10.52 (6.78, 16.33) | 10.52 (6.84, 16.19) | 171.49 | 3.39 (2.77) | 10.47 (7.25) |
| 75 |  | Anion gap increased | 5 | 9.91 (4.12, 23.86) | 9.91 (4.1, 23.94) | 39.9 | 3.3 (2.15) | 9.88 (4.74) |
| 76 |  | Apgar score low | 5 | 9.89 (4.11, 23.81) | 9.89 (4.09, 23.89) | 39.78 | 3.3 (2.14) | 9.85 (4.72) |
| 77 |  | Echocardiogram abnormal | 5 | 9.45 (3.93, 22.76) | 9.45 (3.91, 22.83) | 37.64 | 3.24 (2.08) | 9.42 (4.52) |
| 78 |  | Myocardial necrosis marker increased | 5 | 9.06 (3.76, 21.8) | 9.05 (3.75, 21.86) | 35.69 | 3.17 (2.02) | 9.02 (4.33) |
| 79 |  | Blood ph decreased | 6 | 8.65 (3.88, 19.29) | 8.65 (3.87, 19.32) | 40.46 | 3.11 (2.04) | 8.62 (4.41) |
| 80 |  | Pulmonary arterial pressure increased | 5 | 7.95 (3.31, 19.14) | 7.95 (3.29, 19.21) | 30.3 | 2.99 (1.83) | 7.93 (3.8) |
| 81 |  | Electrocardiogram abnormal | 24 | 7.85 (5.25, 11.72) | 7.84 (5.3, 11.6) | 142.76 | 2.97 (2.4) | 7.82 (5.59) |
| 82 |  | Heart rate increased | 261 | 7.1 (6.28, 8.02) | 7.03 (6.25, 7.91) | 1347.57 | 2.81 (2.63) | 7.01 (6.33) |
| 83 |  | Electrocardiogram qrs complex prolonged | 12 | 6.62 (3.76, 11.67) | 6.62 (3.75, 11.69) | 57.07 | 2.72 (1.94) | 6.6 (4.11) |
| 84 |  | Po2 decreased | 3 | 6.4 (2.06, 19.86) | 6.39 (2.05, 19.92) | 13.62 | 2.67 (1.26) | 6.38 (2.47) |
| 85 |  | Pulse absent | 11 | 5.93 (3.28, 10.72) | 5.93 (3.29, 10.68) | 44.98 | 2.57 (1.75) | 5.92 (3.61) |
| 86 |  | Ejection fraction decreased | 34 | 5.76 (4.11, 8.06) | 5.75 (4.12, 8.02) | 133.1 | 2.52 (2.04) | 5.74 (4.33) |
| 87 |  | Electrocardiogram qt prolonged | 60 | 4.5 (3.49, 5.8) | 4.49 (3.48, 5.79) | 162.52 | 2.16 (1.8) | 4.48 (3.63) |
| 88 |  | Drug screen false positive | 5 | 4.48 (1.86, 10.77) | 4.48 (1.85, 10.82) | 13.48 | 2.16 (1.01) | 4.47 (2.15) |
| 89 |  | Drug screen positive | 11 | 3.74 (2.07, 6.76) | 3.74 (2.08, 6.73) | 22.02 | 1.9 (1.08) | 3.73 (2.28) |
| 90 |  | Intraocular pressure increased | 18 | 3.66 (2.31, 5.82) | 3.66 (2.29, 5.86) | 34.75 | 1.87 (1.22) | 3.66 (2.48) |
| 91 |  | Blood pressure increased | 138 | 2.39 (2.02, 2.83) | 2.38 (2.03, 2.78) | 110.87 | 1.25 (1.01) | 2.38 (2.07) |
| 92 | General disorders and administration site conditions | Injection site ischaemia | 39 | 3242.28 (2004.71, 5243.84) | 3236.49 (1982.76, 5282.98) | 53797.29 | 10.43 (9.87) | 1380.84 (923.49) |
| 93 |  | Injection site pallor | 132 | 2022.81 (1604.77, 2549.73) | 2010.6 (1589.2, 2543.73) | 144453.51 | 10.1 (9.8) | 1095.88 (902.9) |
| 94 |  | Medical device site laceration | 9 | 1547.75 (669.85, 3576.24) | 1547.11 (666.04, 3593.72) | 8464.55 | 9.88 (8.82) | 942.11 (467.48) |
| 95 |  | Injection site anaesthesia | 46 | 400.5 (293.06, 547.33) | 399.66 (292.08, 546.87) | 15687.28 | 8.42 (7.98) | 342.88 (264.02) |
| 96 |  | Application site pallor | 4 | 343.87 (120.6, 980.44) | 343.8 (121.66, 971.52) | 1196.32 | 8.23 (6.88) | 300.95 (125.24) |
| 97 |  | Injection site laceration | 68 | 315.09 (244.64, 405.82) | 314.11 (243.46, 405.27) | 18773.52 | 8.12 (7.76) | 277.96 (224.92) |
| 98 |  | Injection site coldness | 64 | 287.13 (221.5, 372.2) | 286.29 (221.9, 369.37) | 16260.62 | 8 (7.63) | 255.96 (206) |
| 99 |  | Injection site movement impairment | 8 | 183.43 (89.37, 376.47) | 183.36 (88.79, 378.66) | 1348.22 | 7.41 (6.43) | 170.45 (93.39) |
| 100 |  | Injection site hypoaesthesia | 72 | 153.71 (121.08, 195.13) | 153.21 (121.1, 193.84) | 10235.98 | 7.17 (6.83) | 144.1 (118.02) |
| 101 |  | Injection site injury | 109 | 64.05 (52.93, 77.5) | 63.73 (52.39, 77.53) | 6557.41 | 5.96 (5.68) | 62.11 (52.95) |
| 102 |  | Injection site nerve damage | 4 | 45.2 (16.81, 121.56) | 45.19 (16.96, 120.41) | 169.68 | 5.47 (4.19) | 44.38 (19.4) |
| 103 |  | Injection site paraesthesia | 23 | 45.05 (29.82, 68.06) | 45 (29.82, 67.92) | 971.41 | 5.47 (4.88) | 44.19 (31.29) |
| 104 |  | Application site haematoma | 3 | 41.5 (13.25, 129.94) | 41.49 (13.31, 129.32) | 116.54 | 5.35 (3.92) | 40.81 (15.7) |
| 105 |  | Injury associated with device | 245 | 34.98 (30.81, 39.71) | 34.6 (30.76, 38.92) | 7882.46 | 5.09 (4.91) | 34.12 (30.68) |
| 106 |  | Vascular stent thrombosis | 18 | 23.9 (15.02, 38.03) | 23.88 (14.92, 38.22) | 390.74 | 4.56 (3.91) | 23.66 (16.04) |
| 107 |  | Injection site discolouration | 90 | 20.06 (16.3, 24.7) | 19.98 (16.11, 24.79) | 1610.11 | 4.31 (4.01) | 19.83 (16.66) |
| 108 |  | Injection site scar | 17 | 16.7 (10.37, 26.92) | 16.69 (10.43, 26.71) | 249.07 | 4.05 (3.38) | 16.58 (11.12) |
| 109 |  | Injection site indentation | 8 | 12.85 (6.41, 25.74) | 12.84 (6.47, 25.5) | 86.91 | 3.68 (2.73) | 12.78 (7.15) |
| 110 |  | Injection site haematoma | 70 | 11.89 (9.4, 15.04) | 11.85 (9.37, 14.99) | 692.38 | 3.56 (3.22) | 11.8 (9.69) |
| 111 |  | Injection site oedema | 7 | 10.78 (5.13, 22.66) | 10.78 (5.12, 22.7) | 61.82 | 3.42 (2.42) | 10.73 (5.77) |
| 112 |  | Feeling jittery | 72 | 9.85 (7.81, 12.42) | 9.82 (7.76, 12.42) | 568.49 | 3.29 (2.96) | 9.79 (8.06) |
| 113 |  | Administration site pain | 3 | 7.69 (2.48, 23.89) | 7.69 (2.47, 23.97) | 17.4 | 2.94 (1.52) | 7.67 (2.97) |
| 114 |  | Injection site haemorrhage | 186 | 6.47 (5.6, 7.48) | 6.42 (5.6, 7.36) | 850.85 | 2.68 (2.47) | 6.41 (5.68) |
| 115 |  | Injection site necrosis | 5 | 6.31 (2.62, 15.17) | 6.31 (2.61, 15.24) | 22.27 | 2.65 (1.5) | 6.29 (3.02) |
| 116 |  | Therapeutic product ineffective | 10 | 6.13 (3.29, 11.4) | 6.13 (3.27, 11.48) | 42.79 | 2.61 (1.76) | 6.11 (3.64) |
| 117 |  | Paradoxical drug reaction | 8 | 6.06 (3.03, 12.13) | 6.06 (3.05, 12.03) | 33.7 | 2.6 (1.65) | 6.05 (3.38) |
| 118 |  | Crepitations | 7 | 5.94 (2.83, 12.48) | 5.94 (2.82, 12.51) | 28.69 | 2.57 (1.57) | 5.93 (3.19) |
| 119 |  | Brain death | 7 | 5.93 (2.82, 12.44) | 5.92 (2.81, 12.47) | 28.58 | 2.56 (1.56) | 5.91 (3.18) |
| 120 |  | Death neonatal | 3 | 5.72 (1.84, 17.75) | 5.72 (1.84, 17.83) | 11.65 | 2.51 (1.1) | 5.71 (2.21) |
| 121 |  | Therapeutic response delayed | 6 | 5.65 (2.54, 12.6) | 5.65 (2.53, 12.62) | 22.92 | 2.5 (1.43) | 5.64 (2.89) |
| 122 |  | Drug ineffective for unapproved indication | 113 | 5.66 (4.71, 6.82) | 5.64 (4.73, 6.73) | 430.82 | 2.49 (2.23) | 5.63 (4.82) |
| 123 |  | Injection site discomfort | 19 | 5.35 (3.41, 8.39) | 5.35 (3.41, 8.4) | 67 | 2.42 (1.78) | 5.34 (3.66) |
| 124 |  | Potentiating drug interaction | 6 | 5.01 (2.25, 11.16) | 5.01 (2.24, 11.19) | 19.21 | 2.32 (1.25) | 5 (2.56) |
| 125 |  | Injection site bruising | 139 | 4.93 (4.17, 5.82) | 4.9 (4.19, 5.73) | 431.45 | 2.29 (2.05) | 4.89 (4.26) |
| 126 |  | Therapeutic product effect delayed | 7 | 4.54 (2.16, 9.53) | 4.54 (2.16, 9.56) | 19.28 | 2.18 (1.18) | 4.53 (2.44) |
| 127 |  | No adverse event | 279 | 4.38 (3.89, 4.93) | 4.34 (3.86, 4.88) | 717.6 | 2.12 (1.95) | 4.33 (3.92) |
| 128 |  | Injection site pain | 428 | 4.03 (3.66, 4.44) | 3.97 (3.6, 4.38) | 955.15 | 1.99 (1.85) | 3.97 (3.66) |
| 129 |  | Drug ineffective | 1867 | 4.04 (3.85, 4.24) | 3.78 (3.63, 3.93) | 3900.73 | 1.92 (1.85) | 3.78 (3.63) |
| 130 |  | Therapy non-responder | 58 | 3.77 (2.91, 4.88) | 3.76 (2.91, 4.85) | 117.56 | 1.91 (1.54) | 3.76 (3.03) |
| 131 |  | Therapeutic response decreased | 78 | 3.52 (2.82, 4.4) | 3.51 (2.83, 4.35) | 139.93 | 1.81 (1.49) | 3.51 (2.91) |
| 132 |  | Injection site swelling | 88 | 3.23 (2.62, 3.98) | 3.22 (2.6, 3.99) | 134.84 | 1.69 (1.39) | 3.22 (2.7) |
| 133 |  | Therapeutic product effect incomplete | 72 | 2.84 (2.25, 3.58) | 2.83 (2.24, 3.58) | 85.41 | 1.5 (1.17) | 2.83 (2.33) |
| 134 | Cardiac disorders | Systolic anterior motion of mitral valve | 13 | 549.2 (300.64, 1003.28) | 548.88 (298.95, 1007.75) | 5789.13 | 8.8 (7.98) | 447.13 (270.07) |
| 135 |  | Myocardial stunning | 10 | 248.22 (129.44, 476) | 248.11 (129.94, 473.75) | 2231.09 | 7.81 (6.92) | 225.01 (130.5) |
| 136 |  | Stress cardiomyopathy | 300 | 156.84 (139.45, 176.39) | 154.7 (137.54, 174.01) | 43049.14 | 7.18 (7.02) | 145.42 (131.8) |
| 137 |  | Kounis syndrome | 66 | 85.84 (67.13, 109.76) | 85.58 (66.33, 110.42) | 5327.82 | 6.37 (6.02) | 82.68 (67.3) |
| 138 |  | Arteriospasm coronary | 88 | 63.26 (51.17, 78.21) | 63.01 (50.79, 78.17) | 5233.76 | 5.94 (5.64) | 61.43 (51.44) |
| 139 |  | Hyperdynamic left ventricle | 4 | 45.85 (17.05, 123.31) | 45.84 (17.2, 122.14) | 172.17 | 5.49 (4.21) | 45 (19.67) |
| 140 |  | Myocardial necrosis | 4 | 44.37 (16.5, 119.3) | 44.36 (16.65, 118.2) | 166.47 | 5.45 (4.17) | 43.58 (19.05) |
| 141 |  | Ventricular tachyarrhythmia | 4 | 32.97 (12.29, 88.45) | 32.97 (12.37, 87.85) | 122.32 | 5.02 (3.75) | 32.54 (14.25) |
| 142 |  | Systolic dysfunction | 9 | 22.83 (11.84, 44.02) | 22.82 (11.95, 43.57) | 186.05 | 4.5 (3.6) | 22.62 (13.06) |
| 143 |  | Myocardial injury | 5 | 20.72 (8.59, 49.96) | 20.71 (8.57, 50.03) | 93 | 4.36 (3.2) | 20.54 (9.83) |
| 144 |  | Ventricular hypokinesia | 24 | 19.25 (12.88, 28.77) | 19.23 (12.99, 28.46) | 411.44 | 4.25 (3.69) | 19.08 (13.63) |
| 145 |  | Myocardial ischaemia | 83 | 18.28 (14.72, 22.69) | 18.21 (14.68, 22.59) | 1340.43 | 4.18 (3.87) | 18.08 (15.09) |
| 146 |  | Ventricular arrhythmia | 25 | 18.14 (12.23, 26.89) | 18.12 (12.24, 26.82) | 401.3 | 4.17 (3.61) | 17.99 (12.94) |
| 147 |  | Ventricular tachycardia | 104 | 16.77 (13.82, 20.34) | 16.69 (13.72, 20.3) | 1523.9 | 4.05 (3.77) | 16.58 (14.11) |
| 148 |  | Cardiogenic shock | 74 | 14.45 (11.5, 18.17) | 14.41 (11.39, 18.23) | 918.09 | 3.84 (3.51) | 14.33 (11.83) |
| 149 |  | Sinus tachycardia | 75 | 14.01 (11.16, 17.58) | 13.96 (11.03, 17.66) | 897.51 | 3.8 (3.47) | 13.89 (11.48) |
| 150 |  | Coronary artery thrombosis | 11 | 13.79 (7.62, 24.94) | 13.78 (7.65, 24.81) | 129.65 | 3.78 (2.96) | 13.71 (8.35) |
| 151 |  | Left ventricular dysfunction | 32 | 12.95 (9.15, 18.33) | 12.93 (9.09, 18.4) | 350.47 | 3.69 (3.19) | 12.87 (9.62) |
| 152 |  | Ventricular fibrillation | 52 | 12.27 (9.34, 16.12) | 12.25 (9.31, 16.12) | 534.37 | 3.61 (3.22) | 12.19 (9.7) |
| 153 |  | Pulseless electrical activity | 17 | 11.28 (7, 18.17) | 11.27 (7.04, 18.04) | 158.38 | 3.49 (2.82) | 11.22 (7.53) |
| 154 |  | Coronary artery dissection | 3 | 9.33 (3, 28.99) | 9.33 (2.99, 29.08) | 22.22 | 3.22 (1.8) | 9.3 (3.6) |
| 155 |  | Long qt syndrome | 8 | 8.79 (4.39, 17.61) | 8.79 (4.43, 17.45) | 55.04 | 3.13 (2.19) | 8.76 (4.9) |
| 156 |  | Tachyarrhythmia | 8 | 8.26 (4.12, 16.53) | 8.25 (4.15, 16.38) | 50.82 | 3.04 (2.1) | 8.23 (4.6) |
| 157 |  | Pulmonary valve incompetence | 4 | 8.2 (3.07, 21.89) | 8.2 (3.08, 21.85) | 25.2 | 3.03 (1.76) | 8.18 (3.6) |
| 158 |  | Acute myocardial infarction | 94 | 8.16 (6.66, 10) | 8.13 (6.68, 9.89) | 586.37 | 3.02 (2.73) | 8.11 (6.84) |
| 159 |  | Dilatation ventricular | 7 | 8.01 (3.81, 16.81) | 8 (3.8, 16.85) | 42.76 | 3 (1.99) | 7.98 (4.29) |
| 160 |  | Supraventricular tachycardia | 29 | 7.93 (5.5, 11.41) | 7.92 (5.46, 11.49) | 174.7 | 2.98 (2.46) | 7.89 (5.82) |
| 161 |  | Ventricular extrasystoles | 31 | 7.68 (5.4, 10.93) | 7.67 (5.39, 10.91) | 179.34 | 2.94 (2.43) | 7.65 (5.7) |
| 162 |  | Tachycardia | 223 | 6.74 (5.91, 7.7) | 6.69 (5.83, 7.67) | 1076.81 | 2.74 (2.55) | 6.67 (5.97) |
| 163 |  | Acute coronary syndrome | 19 | 5.83 (3.71, 9.14) | 5.82 (3.71, 9.13) | 75.72 | 2.54 (1.91) | 5.81 (3.99) |
| 164 |  | Cardiac arrest | 177 | 5.65 (4.87, 6.55) | 5.61 (4.89, 6.43) | 669.64 | 2.48 (2.27) | 5.6 (4.95) |
| 165 |  | Ventricular dysfunction | 5 | 5.36 (2.23, 12.88) | 5.36 (2.22, 12.95) | 17.67 | 2.42 (1.26) | 5.35 (2.56) |
| 166 |  | Nodal rhythm | 3 | 5.24 (1.69, 16.27) | 5.24 (1.68, 16.33) | 10.27 | 2.39 (0.97) | 5.23 (2.03) |
| 167 |  | Cardiopulmonary failure | 8 | 5.22 (2.61, 10.45) | 5.22 (2.63, 10.37) | 27.24 | 2.38 (1.44) | 5.21 (2.92) |
| 168 |  | Palpitations | 223 | 5.11 (4.48, 5.83) | 5.07 (4.42, 5.82) | 728.44 | 2.34 (2.15) | 5.06 (4.53) |
| 169 |  | Bradycardia | 103 | 5.06 (4.17, 6.14) | 5.04 (4.14, 6.13) | 333.04 | 2.33 (2.05) | 5.03 (4.28) |
| 170 |  | Cardiomyopathy | 28 | 4.93 (3.4, 7.14) | 4.92 (3.39, 7.14) | 87.31 | 2.3 (1.77) | 4.91 (3.6) |
| 171 |  | Torsade de pointes | 12 | 4.05 (2.3, 7.13) | 4.05 (2.29, 7.15) | 27.47 | 2.01 (1.23) | 4.04 (2.52) |
| 172 |  | Coronary artery stenosis | 8 | 3.96 (1.98, 7.92) | 3.96 (1.99, 7.86) | 17.66 | 1.98 (1.04) | 3.95 (2.21) |
| 173 |  | Cardiac failure acute | 9 | 3.7 (1.92, 7.11) | 3.7 (1.94, 7.06) | 17.68 | 1.88 (0.99) | 3.69 (2.14) |
| 174 |  | Mitral valve incompetence | 14 | 3.21 (1.9, 5.42) | 3.21 (1.89, 5.45) | 21.25 | 1.68 (0.95) | 3.2 (2.07) |
| 175 | Vascular disorders | Vasoconstriction | 33 | 112.02 (79, 158.86) | 111.86 (78.61, 159.18) | 3464.6 | 6.74 (6.24) | 106.93 (79.83) |
| 176 |  | Diastolic hypertension | 4 | 83 (30.63, 224.91) | 82.99 (30.54, 225.5) | 313.2 | 6.33 (5.04) | 80.25 (34.85) |
| 177 |  | Dry gangrene | 11 | 45.35 (24.97, 82.36) | 45.33 (25.18, 81.61) | 468.06 | 5.48 (4.65) | 44.51 (27.02) |
| 178 |  | Vasospasm | 14 | 41.27 (24.33, 70) | 41.24 (24.29, 70.01) | 540.44 | 5.34 (4.61) | 40.56 (26.07) |
| 179 |  | Systolic hypertension | 6 | 35.75 (15.96, 80.06) | 35.74 (16, 79.83) | 199.66 | 5.14 (4.06) | 35.23 (17.95) |
| 180 |  | Extremity necrosis | 21 | 32.06 (20.84, 49.32) | 32.03 (20.81, 49.3) | 622.96 | 4.98 (4.38) | 31.62 (22.05) |
| 181 |  | Peripheral ischaemia | 41 | 26.65 (19.59, 36.27) | 26.6 (19.44, 36.4) | 999.3 | 4.72 (4.28) | 26.32 (20.34) |
| 182 |  | Air embolism | 3 | 17.15 (5.51, 53.4) | 17.15 (5.5, 53.45) | 45.3 | 4.09 (2.67) | 17.04 (6.59) |
| 183 |  | Haemodynamic instability | 26 | 9.59 (6.52, 14.1) | 9.58 (6.47, 14.18) | 199.08 | 3.26 (2.71) | 9.55 (6.92) |
| 184 |  | Distributive shock | 5 | 7.92 (3.29, 19.07) | 7.92 (3.28, 19.13) | 30.14 | 2.98 (1.82) | 7.9 (3.79) |
| 185 |  | Hypoperfusion | 3 | 7.71 (2.48, 23.94) | 7.71 (2.47, 24.03) | 17.45 | 2.94 (1.52) | 7.68 (2.98) |
| 186 |  | Hypertensive emergency | 3 | 6.98 (2.25, 21.67) | 6.98 (2.24, 21.76) | 15.31 | 2.8 (1.38) | 6.96 (2.7) |
| 187 |  | Poor peripheral circulation | 11 | 6.68 (3.69, 12.07) | 6.67 (3.7, 12.01) | 52.93 | 2.74 (1.92) | 6.66 (4.06) |
| 188 |  | Vasodilatation | 6 | 6.27 (2.82, 13.98) | 6.27 (2.81, 14) | 26.52 | 2.65 (1.58) | 6.26 (3.2) |
| 189 |  | Ischaemia | 11 | 6.09 (3.37, 11) | 6.08 (3.38, 10.95) | 46.62 | 2.6 (1.78) | 6.07 (3.7) |
| 190 |  | Pallor | 53 | 5.08 (3.88, 6.66) | 5.07 (3.85, 6.67) | 173.04 | 2.34 (1.95) | 5.06 (4.04) |
| 191 |  | Hypertensive crisis | 21 | 4.95 (3.23, 7.6) | 4.95 (3.22, 7.62) | 66 | 2.3 (1.7) | 4.94 (3.45) |
| 192 |  | Shock | 36 | 4.29 (3.09, 5.95) | 4.28 (3.07, 5.97) | 90.4 | 2.1 (1.63) | 4.28 (3.25) |
| 193 |  | Circulatory collapse | 24 | 3.63 (2.43, 5.42) | 3.63 (2.45, 5.37) | 45.58 | 1.86 (1.29) | 3.62 (2.59) |
| 194 |  | Cyanosis | 20 | 3.32 (2.14, 5.15) | 3.32 (2.16, 5.11) | 32.37 | 1.73 (1.11) | 3.32 (2.3) |
| 195 |  | Peripheral coldness | 17 | 3.27 (2.03, 5.26) | 3.26 (2.04, 5.22) | 26.67 | 1.71 (1.04) | 3.26 (2.19) |
| 196 |  | Hypotension | 218 | 2.9 (2.54, 3.32) | 2.88 (2.51, 3.3) | 268.53 | 1.53 (1.33) | 2.88 (2.58) |
| 197 |  | Hypertension | 194 | 2.44 (2.12, 2.81) | 2.43 (2.12, 2.79) | 163.71 | 1.28 (1.08) | 2.43 (2.16) |
| 198 | Respiratory, thoracic and mediastinal disorders | Respiratory tract irritation | 5 | 13.2 (5.48, 31.79) | 13.19 (5.46, 31.86) | 56.04 | 3.71 (2.56) | 13.13 (6.29) |
| 199 |  | Grunting | 3 | 12.96 (4.17, 40.32) | 12.96 (4.16, 40.39) | 32.94 | 3.69 (2.27) | 12.9 (4.99) |
| 200 |  | Acute pulmonary oedema | 25 | 11.67 (7.87, 17.29) | 11.66 (7.88, 17.26) | 242.38 | 3.54 (2.98) | 11.6 (8.35) |
| 201 |  | Nasal inflammation | 3 | 8.09 (2.6, 25.12) | 8.08 (2.59, 25.18) | 18.56 | 3.01 (1.59) | 8.06 (3.12) |
| 202 |  | Stridor | 7 | 6.32 (3.01, 13.27) | 6.32 (3, 13.31) | 31.26 | 2.66 (1.66) | 6.31 (3.39) |
| 203 |  | Pulmonary oedema | 102 | 6.01 (4.95, 7.3) | 5.99 (4.92, 7.29) | 422.82 | 2.58 (2.3) | 5.97 (5.07) |
| 204 |  | Hyperventilation | 13 | 5.83 (3.38, 10.06) | 5.83 (3.37, 10.09) | 51.92 | 2.54 (1.78) | 5.82 (3.69) |
| 205 |  | Respiratory acidosis | 8 | 5.48 (2.74, 10.96) | 5.47 (2.75, 10.86) | 29.19 | 2.45 (1.51) | 5.46 (3.06) |
| 206 |  | Tachypnoea | 21 | 4.19 (2.73, 6.43) | 4.19 (2.72, 6.45) | 50.88 | 2.06 (1.46) | 4.18 (2.92) |
| 207 |  | Pharyngeal oedema | 25 | 3.93 (2.66, 5.83) | 3.93 (2.66, 5.82) | 54.55 | 1.97 (1.42) | 3.93 (2.83) |
| 208 |  | Sputum discoloured | 13 | 3.7 (2.14, 6.37) | 3.69 (2.13, 6.39) | 25.5 | 1.88 (1.13) | 3.69 (2.34) |
| 209 |  | Respiratory distress | 33 | 3.16 (2.24, 4.45) | 3.16 (2.26, 4.41) | 48.55 | 1.66 (1.17) | 3.15 (2.37) |
| 210 |  | Hypoxia | 37 | 2.84 (2.05, 3.91) | 2.83 (2.07, 3.87) | 43.82 | 1.5 (1.04) | 2.83 (2.16) |
| 211 | Nervous system disorders | Harlequin syndrome | 3 | 124.5 (39.01, 397.37) | 124.48 (39.16, 395.66) | 349.39 | 6.89 (5.43) | 118.41 (44.84) |
| 212 |  | Cerebral vasoconstriction | 5 | 17.19 (7.13, 41.44) | 17.19 (7.12, 41.53) | 75.7 | 4.09 (2.93) | 17.08 (8.18) |
| 213 |  | Reversible cerebral vasoconstriction syndrome | 11 | 16.95 (9.36, 30.67) | 16.94 (9.41, 30.5) | 163.81 | 4.07 (3.25) | 16.83 (10.24) |
| 214 |  | Horner's syndrome | 3 | 15.07 (4.84, 46.91) | 15.07 (4.84, 46.97) | 39.17 | 3.91 (2.49) | 14.99 (5.8) |
| 215 |  | Hypoxic-ischaemic encephalopathy | 10 | 10.17 (5.46, 18.93) | 10.17 (5.43, 19.04) | 82.31 | 3.34 (2.49) | 10.13 (6.02) |
| 216 |  | Brain injury | 28 | 7.44 (5.13, 10.78) | 7.43 (5.12, 10.78) | 155.3 | 2.89 (2.36) | 7.41 (5.43) |
| 217 |  | Tunnel vision | 4 | 5.77 (2.16, 15.4) | 5.77 (2.17, 15.37) | 15.74 | 2.53 (1.26) | 5.76 (2.53) |
| 218 |  | Sensory loss | 11 | 3.59 (1.99, 6.49) | 3.59 (1.99, 6.46) | 20.56 | 1.84 (1.03) | 3.59 (2.19) |
| 219 |  | Tremor | 211 | 3.35 (2.93, 3.84) | 3.33 (2.9, 3.82) | 344.06 | 1.73 (1.54) | 3.32 (2.97) |
| 220 |  | Paralysis | 17 | 3.1 (1.93, 4.99) | 3.1 (1.94, 4.96) | 24.16 | 1.63 (0.96) | 3.1 (2.08) |
| 221 |  | Loss of consciousness | 141 | 2.92 (2.47, 3.44) | 2.9 (2.48, 3.39) | 176.07 | 1.54 (1.3) | 2.9 (2.53) |
| 222 | Eye disorders | Anterior chamber disorder | 3 | 35.57 (11.38, 111.22) | 35.57 (11.41, 110.86) | 99.31 | 5.13 (3.7) | 35.06 (13.51) |
| 223 |  | Corneal oedema | 24 | 28.41 (19, 42.5) | 28.38 (18.8, 42.83) | 626.67 | 4.81 (4.24) | 28.06 (20.04) |
| 224 |  | Lagophthalmos | 3 | 19.2 (6.17, 59.82) | 19.2 (6.16, 59.84) | 51.35 | 4.25 (2.83) | 19.06 (7.37) |
| 225 |  | Retinal exudates | 4 | 11.07 (4.14, 29.56) | 11.06 (4.15, 29.47) | 36.45 | 3.46 (2.19) | 11.02 (4.84) |
| 226 |  | Retinal artery occlusion | 10 | 10.85 (5.83, 20.19) | 10.84 (5.79, 20.3) | 88.93 | 3.43 (2.58) | 10.8 (6.42) |
| 227 |  | Pupil fixed | 7 | 10.26 (4.88, 21.56) | 10.26 (4.87, 21.61) | 58.25 | 3.35 (2.35) | 10.22 (5.49) |
| 228 |  | Amaurosis fugax | 3 | 7.73 (2.49, 24.02) | 7.73 (2.48, 24.09) | 17.52 | 2.95 (1.53) | 7.71 (2.99) |
| 229 |  | Pupillary reflex impaired | 4 | 5.82 (2.18, 15.52) | 5.82 (2.18, 15.51) | 15.92 | 2.54 (1.27) | 5.8 (2.55) |
| 230 |  | Mydriasis | 19 | 3.97 (2.53, 6.22) | 3.96 (2.52, 6.22) | 42.04 | 1.98 (1.35) | 3.96 (2.72) |
| 231 | Immune system disorders | Allergy to arthropod sting | 3 | 18.01 (5.78, 56.07) | 18 (5.78, 56.1) | 47.82 | 4.16 (2.74) | 17.88 (6.91) |
| 232 |  | Reaction to preservatives | 5 | 16.49 (6.84, 39.73) | 16.48 (6.82, 39.81) | 72.23 | 4.03 (2.87) | 16.38 (7.85) |
| 233 |  | Perfume sensitivity | 3 | 15.53 (4.99, 48.33) | 15.53 (4.98, 48.4) | 40.51 | 3.95 (2.53) | 15.43 (5.97) |
| 234 |  | Sensitisation | 3 | 9.93 (3.2, 30.87) | 9.93 (3.19, 30.95) | 24 | 3.31 (1.89) | 9.89 (3.83) |
| 235 |  | Anaphylactic reaction | 183 | 9.32 (8.06, 10.78) | 9.25 (8.06, 10.61) | 1342.88 | 3.2 (3) | 9.22 (8.16) |
| 236 |  | Drug hypersensitivity | 668 | 9.23 (8.54, 9.97) | 8.98 (8.3, 9.71) | 4734.36 | 3.16 (3.05) | 8.95 (8.39) |
| 237 |  | Allergy to chemicals | 3 | 6.61 (2.13, 20.52) | 6.61 (2.12, 20.6) | 14.23 | 2.72 (1.3) | 6.59 (2.55) |
| 238 |  | Anaphylactic shock | 59 | 6.34 (4.91, 8.19) | 6.33 (4.91, 8.17) | 264.01 | 2.66 (2.29) | 6.31 (5.1) |
| 239 | Infections and infestations | Gas gangrene | 10 | 78.43 (41.77, 147.25) | 78.39 (41.87, 146.77) | 739.95 | 6.25 (5.38) | 75.95 (44.83) |
| 240 |  | Eye infection staphylococcal | 3 | 26.74 (8.57, 83.45) | 26.74 (8.58, 83.34) | 73.52 | 4.73 (3.3) | 26.46 (10.21) |
| 241 |  | Gangrene | 23 | 9.78 (6.49, 14.74) | 9.77 (6.47, 14.75) | 180.45 | 3.28 (2.71) | 9.74 (6.91) |
| 242 |  | Endophthalmitis | 20 | 8.95 (5.77, 13.88) | 8.94 (5.81, 13.76) | 140.53 | 3.16 (2.54) | 8.91 (6.17) |
| 243 |  | Clostridial infection | 11 | 8.01 (4.43, 14.49) | 8.01 (4.45, 14.42) | 67.26 | 3 (2.18) | 7.99 (4.87) |
| 244 | Gastrointestinal disorders | Intestinal haematoma | 4 | 64.19 (23.78, 173.28) | 64.18 (23.62, 174.39) | 242.31 | 5.97 (4.68) | 62.54 (27.24) |
| 245 |  | Gastrointestinal ischaemia | 5 | 46.83 (19.33, 113.49) | 46.82 (19.38, 113.1) | 219.94 | 5.52 (4.35) | 45.95 (21.91) |
| 246 |  | Duodenal obstruction | 3 | 27.15 (8.7, 84.71) | 27.14 (8.71, 84.59) | 74.7 | 4.75 (3.32) | 26.85 (10.36) |
| 247 |  | Intestinal ischaemia | 12 | 5.86 (3.32, 10.32) | 5.86 (3.32, 10.35) | 48.2 | 2.55 (1.76) | 5.84 (3.64) |
| 248 |  | Paraesthesia oral | 17 | 3.16 (1.97, 5.09) | 3.16 (1.97, 5.06) | 25.11 | 1.66 (0.99) | 3.16 (2.12) |
| 249 | Musculoskeletal and connective tissue disorders | Chondrolysis | 10 | 52.46 (28.03, 98.16) | 52.43 (28, 98.17) | 493.76 | 5.68 (4.82) | 51.34 (30.39) |
| 250 |  | Compartment syndrome | 11 | 11.08 (6.13, 20.03) | 11.07 (6.15, 19.93) | 100.33 | 3.46 (2.64) | 11.03 (6.72) |
| 251 |  | Necrotising myositis | 3 | 11.02 (3.55, 34.27) | 11.02 (3.54, 34.35) | 27.22 | 3.46 (2.04) | 10.98 (4.25) |
| 252 |  | Ligament disorder | 3 | 8.44 (2.71, 26.21) | 8.43 (2.7, 26.27) | 19.59 | 3.07 (1.65) | 8.41 (3.26) |
| 253 | Skin and subcutaneous tissue disorders | Keloid scar | 3 | 15.6 (5.01, 48.54) | 15.59 (5, 48.59) | 40.71 | 3.95 (2.53) | 15.5 (5.99) |
| 254 |  | Skin necrosis | 13 | 6.53 (3.79, 11.25) | 6.52 (3.77, 11.29) | 60.62 | 2.7 (1.94) | 6.51 (4.13) |
| 255 |  | Angioedema | 49 | 2.98 (2.25, 3.94) | 2.97 (2.26, 3.91) | 64.1 | 1.57 (1.17) | 2.97 (2.35) |
| 256 | Psychiatric disorders | Near death experience | 5 | 4.53 (1.88, 10.89) | 4.53 (1.88, 10.94) | 13.71 | 2.18 (1.02) | 4.52 (2.17) |
| 257 |  | Post-traumatic stress disorder | 9 | 3.6 (1.87, 6.92) | 3.6 (1.89, 6.87) | 16.86 | 1.85 (0.95) | 3.59 (2.08) |
| 258 |  | Nervousness | 65 | 3.18 (2.49, 4.06) | 3.18 (2.51, 4.02) | 96.83 | 1.67 (1.32) | 3.17 (2.59) |
| 259 | Metabolism and nutrition disorders | Metabolic acidosis | 49 | 4.21 (3.18, 5.57) | 4.2 (3.19, 5.53) | 119.38 | 2.07 (1.67) | 4.2 (3.32) |
| 260 |  | Lactic acidosis | 41 | 3.54 (2.61, 4.81) | 3.54 (2.59, 4.84) | 74.51 | 1.82 (1.38) | 3.53 (2.73) |
| 261 | Congenital, familial and genetic disorders | Left ventricle outflow tract obstruction | 22 | 179.66 (116.47, 277.11) | 179.48 (116.61, 276.24) | 3633.65 | 7.38 (6.77) | 167.09 (116.27) |
| 262 |  | Brugada syndrome | 3 | 8.12 (2.61, 25.23) | 8.12 (2.61, 25.31) | 18.67 | 3.02 (1.6) | 8.1 (3.14) |
| 263 | Pregnancy, puerperium and perinatal conditions | Uterine hypertonus | 4 | 37.46 (13.95, 100.59) | 37.46 (14.06, 99.81) | 139.76 | 5.21 (3.93) | 36.9 (16.15) |
| 264 | Endocrine disorders | Thyrotoxic crisis | 5 | 13.14 (5.46, 31.65) | 13.14 (5.44, 31.74) | 55.76 | 3.71 (2.55) | 13.07 (6.26) |

Abbreviations: ROR, reporting odds ratio; PRR, proportional reporting ratio; IC, information component; EBGM, Empirical Bayes Geometric Mean.

Table S4 Top 50 signal strength of adverse events of subcutaneous epinephrine ranked by EBGM at the preferred term level in FDA adverse event reporting system database.

| Rank | System organ class | Preferred term | Case reports | ROR (95% CI) | PRR (95% CI) | χ^2^ | IC (IC025) | EBGM (EBGM05) |
| --- | --- | --- | --- | --- | --- | --- | --- | --- |
| 1 | Cardiac disorders | Stress cardiomyopathy | 25 | 343.6 (230.56, 512.05) | 333.46 (225.32, 493.5) | 8243.16 | 8.37 (7.81) | 331.69 (237.55) |
| 2 |  | Arteriospasm coronary | 8 | 149.58 (74.49, 300.34) | 148.17 (74.62, 294.23) | 1166.72 | 7.21 (6.26) | 147.82 (82.49) |
| 3 |  | Tachyarrhythmia | 4 | 107.23 (40.12, 286.62) | 106.73 (40.06, 284.38) | 418.26 | 6.74 (5.47) | 106.55 (46.8) |
| 4 |  | Ventricular hypokinesia | 4 | 83.28 (31.16, 222.56) | 82.89 (31.11, 220.86) | 323.2 | 6.37 (5.1) | 82.78 (36.37) |
| 5 |  | Sinus tachycardia | 12 | 58.6 (33.14, 103.63) | 57.78 (32.73, 102.01) | 669.13 | 5.85 (5.06) | 57.73 (35.83) |
| 6 |  | Ventricular arrhythmia | 3 | 56.43 (18.15, 175.41) | 56.23 (18.04, 175.26) | 162.62 | 5.81 (4.39) | 56.18 (21.75) |
| 7 |  | Myocardial ischaemia | 5 | 28.54 (11.85, 68.77) | 28.38 (11.75, 68.56) | 132.05 | 4.83 (3.67) | 28.37 (13.59) |
| 8 |  | Cardiotoxicity | 3 | 24.78 (7.97, 77.01) | 24.7 (7.92, 76.98) | 68.19 | 4.63 (3.21) | 24.69 (9.56) |
| 9 |  | Supraventricular tachycardia | 3 | 21.26 (6.84, 66.05) | 21.18 (6.8, 66.01) | 57.68 | 4.4 (2.99) | 21.18 (8.2) |
| 10 |  | Acute myocardial infarction | 6 | 13.52 (6.05, 30.18) | 13.43 (6.01, 30) | 69.03 | 3.75 (2.67) | 13.42 (6.86) |
| 11 |  | Tachycardia | 10 | 7.84 (4.2, 14.62) | 7.75 (4.22, 14.23) | 58.91 | 2.95 (2.1) | 7.75 (4.6) |
| 12 |  | Cardiac failure | 5 | 4.27 (1.77, 10.29) | 4.25 (1.76, 10.27) | 12.45 | 2.09 (0.93) | 4.25 (2.04) |
| 13 |  | Myocardial infarction | 10 | 3.75 (2.01, 7) | 3.72 (2.03, 6.83) | 19.96 | 1.9 (1.04) | 3.72 (2.21) |
| 14 | Injury, poisoning and procedural complications | Product packaging confusion | 4 | 113.77 (42.56, 304.11) | 113.23 (42.5, 301.7) | 444.18 | 6.82 (5.55) | 113.03 (49.65) |
| 15 |  | Accidental exposure to product by child | 10 | 106.4 (57.01, 198.59) | 105.15 (57.27, 193.06) | 1030 | 6.71 (5.86) | 104.98 (62.28) |
| 16 |  | Product label confusion | 3 | 38.58 (12.41, 119.89) | 38.44 (12.33, 119.81) | 109.35 | 5.26 (3.85) | 38.42 (14.88) |
| 17 |  | Accidental overdose | 16 | 32.24 (19.66, 52.89) | 31.65 (19.39, 51.66) | 474.99 | 4.98 (4.29) | 31.64 (20.91) |
| 18 |  | Accidental exposure to product | 33 | 27.18 (19.19, 38.5) | 26.16 (18.75, 36.5) | 799.27 | 4.71 (4.21) | 26.15 (19.54) |
| 19 |  | Expired product administered | 9 | 19.12 (9.91, 36.88) | 18.93 (9.91, 36.15) | 152.85 | 4.24 (3.34) | 18.92 (10.92) |
| 20 |  | Incorrect dose administered by device | 5 | 16.2 (6.72, 39.02) | 16.11 (6.67, 38.92) | 70.84 | 4.01 (2.85) | 16.1 (7.72) |
| 21 |  | Medication error | 10 | 12.65 (6.78, 23.61) | 12.52 (6.82, 22.99) | 106.04 | 3.65 (2.79) | 12.51 (7.43) |
| 22 |  | Circumstance or information capable of leading to medication error | 3 | 6.29 (2.02, 19.54) | 6.27 (2.01, 19.54) | 13.3 | 2.65 (1.23) | 6.27 (2.43) |
| 23 |  | Product storage error | 7 | 6.26 (2.97, 13.16) | 6.21 (2.95, 13.08) | 30.65 | 2.64 (1.63) | 6.21 (3.33) |
| 24 | General disorders and administration site conditions | Injection site ischaemia | 4 | 8627.09 (3026.24, 24593.78) | 8586.26 (3038.5, 24263.26) | 30174.98 | 12.88 (11.52) | 7545.62 (3140.6) |
| 25 |  | Injection site pallor | 12 | 4795.97 (2656.38, 8658.9) | 4727.87 (2626.03, 8511.98) | 52707.58 | 12.1 (11.28) | 4394.21 (2680.32) |
| 26 |  | Injection site coldness | 5 | 581.97 (240.62, 1407.57) | 578.53 (239.48, 1397.57) | 2856.17 | 9.16 (8) | 573.22 (273.76) |
| 27 |  | Injection site hypoaesthesia | 7 | 388.49 (184.21, 819.3) | 385.28 (182.94, 811.41) | 2666.54 | 8.58 (7.57) | 382.92 (205.09) |
| 28 |  | Injury associated with device | 7 | 25.77 (12.25, 54.24) | 25.57 (12.14, 53.85) | 165.23 | 4.68 (3.67) | 25.56 (13.71) |
| 29 |  | Drug ineffective for unapproved indication | 12 | 15.7 (8.88, 27.77) | 15.49 (8.77, 27.35) | 162.81 | 3.95 (3.16) | 15.49 (9.62) |
| 30 |  | No adverse event | 15 | 6.12 (3.68, 10.21) | 6.03 (3.62, 10.04) | 63.17 | 2.59 (1.88) | 6.03 (3.94) |
| 31 |  | Injection site pain | 17 | 4.14 (2.56, 6.7) | 4.08 (2.55, 6.53) | 39.74 | 2.03 (1.36) | 4.08 (2.73) |
| 32 | Vascular disorders | Vasoconstriction | 4 | 352.37 (131.58, 943.65) | 350.71 (131.63, 934.45) | 1387.04 | 8.45 (7.17) | 348.75 (152.95) |
| 33 |  | Peripheral ischaemia | 4 | 67.45 (25.24, 180.23) | 67.13 (25.19, 178.86) | 260.33 | 6.07 (4.8) | 67.06 (29.47) |
| 34 |  | Hypertension | 13 | 4.26 (2.46, 7.37) | 4.21 (2.43, 7.29) | 31.96 | 2.07 (1.31) | 4.21 (2.66) |
| 35 |  | Hypotension | 12 | 4.15 (2.35, 7.33) | 4.1 (2.32, 7.24) | 28.26 | 2.04 (1.25) | 4.1 (2.55) |
| 36 | Investigations | Electrocardiogram abnormal | 5 | 42.49 (17.63, 102.38) | 42.24 (17.49, 102.04) | 201.23 | 5.4 (4.24) | 42.22 (20.23) |
| 37 |  | Ejection fraction decreased | 3 | 13.16 (4.24, 40.9) | 13.12 (4.21, 40.89) | 33.59 | 3.71 (2.3) | 13.12 (5.08) |
| 38 |  | Oxygen saturation decreased | 7 | 8.98 (4.27, 18.89) | 8.91 (4.23, 18.76) | 49.21 | 3.16 (2.15) | 8.91 (4.78) |
| 39 |  | Blood pressure increased | 9 | 4.05 (2.1, 7.81) | 4.02 (2.11, 7.68) | 20.46 | 2.01 (1.11) | 4.02 (2.32) |
| 40 | Respiratory, thoracic and mediastinal disorders | Acute pulmonary oedema | 3 | 36.3 (11.68, 112.83) | 36.18 (11.61, 112.77) | 102.57 | 5.18 (3.76) | 36.16 (14) |
| 41 |  | Pulmonary oedema | 11 | 16.9 (9.32, 30.64) | 16.7 (9.28, 30.07) | 162.4 | 4.06 (3.24) | 16.69 (10.15) |
| 42 |  | Respiratory distress | 3 | 7.44 (2.4, 23.13) | 7.42 (2.38, 23.13) | 16.67 | 2.89 (1.47) | 7.42 (2.87) |
| 43 | Nervous system disorders | Subarachnoid haemorrhage | 3 | 19.92 (6.41, 61.9) | 19.85 (6.37, 61.87) | 53.7 | 4.31 (2.89) | 19.85 (7.69) |
| 44 |  | Ischaemic stroke | 5 | 19.16 (7.95, 46.15) | 19.05 (7.89, 46.02) | 85.51 | 4.25 (3.09) | 19.04 (9.13) |
| 45 |  | Loss of consciousness | 8 | 4.29 (2.14, 8.61) | 4.26 (2.15, 8.46) | 20 | 2.09 (1.14) | 4.26 (2.38) |
| 46 | Immune system disorders | Anaphylactic shock | 5 | 13.94 (5.79, 33.59) | 13.87 (5.74, 33.51) | 59.71 | 3.79 (2.63) | 13.87 (6.64) |
| 47 |  | Anaphylactic reaction | 7 | 9.22 (4.38, 19.4) | 9.15 (4.34, 19.27) | 50.88 | 3.19 (2.19) | 9.15 (4.91) |
| 48 | Metabolism and nutrition disorders | Lactic acidosis | 4 | 8.96 (3.36, 23.94) | 8.92 (3.35, 23.77) | 28.16 | 3.16 (1.89) | 8.92 (3.92) |
| 49 | Eye disorders | Retinal artery occlusion | 3 | 84.42 (27.15, 262.48) | 84.12 (26.99, 262.18) | 246.08 | 6.39 (4.97) | 84.01 (32.52) |
| 50 | Endocrine disorders | Thyrotoxic crisis | 3 | 204.6 (65.73, 636.84) | 203.88 (65.41, 635.45) | 603.68 | 7.67 (6.25) | 203.21 (78.58) |

Abbreviations: ROR, reporting odds ratio; PRR, proportional reporting ratio; IC, information component; EBGM, Empirical Bayes Geometric Mean.

Table S5 Top 50 signal strength of adverse events of intravenous epinephrine ranked by EBGM at the preferred term level in FDA adverse event reporting system database.

| Rank | System organ class | Preferred term | Case reports | ROR (95% CI) | PRR (95% CI) | χ^2^ | IC (IC025) | EBGM (EBGM05) |
| --- | --- | --- | --- | --- | --- | --- | --- | --- |
| 1 | Cardiac disorders | Systolic anterior motion of mitral valve | 4 | 1747.79 (633.59, 4821.34) | 1744.49 (629.56, 4833.94) | 6512.92 | 10.67 (9.35) | 1630.16 (697.43) |
| 2 |  | Myocardial stunning | 6 | 1542.04 (675.34, 3521.02) | 1537.67 (675.07, 3502.47) | 8677.29 | 10.5 (9.39) | 1448.15 (725.74) |
| 3 |  | Stress cardiomyopathy | 130 | 737.71 (616.35, 882.98) | 692.45 (580.47, 826.03) | 87334.16 | 9.4 (9.14) | 673.71 (579.64) |
| 4 |  | Myocardial necrosis | 3 | 344.16 (110.05, 1076.32) | 343.67 (110.26, 1071.14) | 1011.05 | 8.41 (6.98) | 339 (130.58) |
| 5 |  | Arteriospasm coronary | 31 | 232.68 (162.95, 332.26) | 229.29 (161.13, 326.29) | 6982.03 | 7.83 (7.32) | 227.2 (168.64) |
| 6 |  | Kounis syndrome | 12 | 161.64 (91.48, 285.6) | 160.73 (91.04, 283.76) | 1892.62 | 7.32 (6.53) | 159.7 (99.19) |
| 7 |  | Systolic dysfunction | 4 | 104.98 (39.28, 280.54) | 104.78 (39.33, 279.18) | 409.44 | 6.71 (5.44) | 104.34 (45.84) |
| 8 |  | Coronary artery dissection | 3 | 96.49 (31.03, 300.07) | 96.35 (30.91, 300.3) | 282 | 6.58 (5.17) | 95.98 (37.14) |
| 9 |  | Ventricular hypokinesia | 10 | 83.14 (44.62, 154.91) | 82.75 (44.2, 154.94) | 805.02 | 6.37 (5.51) | 82.48 (49) |
| 10 |  | Myocardial ischaemia | 34 | 78.31 (55.77, 109.95) | 77.07 (55.23, 107.55) | 2545.39 | 6.26 (5.78) | 76.83 (57.84) |
| 11 |  | Ventricular tachycardia | 46 | 77.93 (58.16, 104.42) | 76.25 (56.83, 102.31) | 3406.84 | 6.25 (5.83) | 76.02 (59.51) |
| 12 |  | Ventricular arrhythmia | 10 | 75.2 (40.37, 140.11) | 74.85 (39.98, 140.15) | 726.53 | 6.22 (5.37) | 74.63 (44.34) |
| 13 |  | Cardiogenic shock | 32 | 65.33 (46.06, 92.67) | 64.36 (45.23, 91.59) | 1991.32 | 6 (5.51) | 64.2 (47.92) |
| 14 |  | Ventricular fibrillation | 21 | 51.58 (33.55, 79.32) | 51.08 (33.19, 78.62) | 1029.18 | 5.67 (5.07) | 50.98 (35.56) |
| 15 |  | Sinus tachycardia | 26 | 50.6 (34.36, 74.53) | 49.99 (33.78, 73.98) | 1246.19 | 5.64 (5.09) | 49.9 (36.09) |
| 16 |  | Ventricular dysfunction | 4 | 44.33 (16.61, 118.34) | 44.25 (16.61, 117.9) | 168.81 | 5.47 (4.2) | 44.18 (19.43) |
| 17 |  | Left ventricular dysfunction | 10 | 41.94 (22.52, 78.1) | 41.74 (22.29, 78.15) | 397.06 | 5.38 (4.53) | 41.68 (24.77) |
| 18 |  | Pulseless electrical activity | 5 | 34.32 (14.26, 82.59) | 34.24 (14.17, 82.71) | 161.14 | 5.1 (3.94) | 34.2 (16.4) |
| 19 |  | Ventricular extrasystoles | 11 | 28.26 (15.62, 51.13) | 28.12 (15.62, 50.63) | 287.45 | 4.81 (3.99) | 28.09 (17.11) |
| 20 |  | Acute myocardial infarction | 28 | 25.35 (17.45, 36.81) | 25.02 (17.24, 36.31) | 645.47 | 4.64 (4.11) | 25 (18.3) |
| 21 |  | Cardiac failure acute | 5 | 21.26 (8.84, 51.15) | 21.21 (8.78, 51.24) | 96.22 | 4.41 (3.25) | 21.19 (10.17) |
| 22 |  | Cardiopulmonary failure | 3 | 20.25 (6.52, 62.86) | 20.22 (6.49, 63.02) | 54.77 | 4.34 (2.92) | 20.21 (7.83) |
| 23 | Investigations | Electrocardiogram st segment depression | 17 | 208.22 (128.94, 336.24) | 206.55 (129.04, 330.61) | 3448.92 | 7.68 (7.01) | 204.86 (137.18) |
| 24 |  | Electrocardiogram st segment elevation | 18 | 147.57 (92.67, 235.01) | 146.32 (91.41, 234.2) | 2582.92 | 7.18 (6.53) | 145.47 (98.56) |
| 25 |  | Electrocardiogram st-t change | 3 | 108.39 (34.84, 337.18) | 108.24 (34.73, 337.36) | 317.37 | 6.75 (5.33) | 107.77 (41.7) |
| 26 |  | Troponin t increased | 4 | 89.19 (33.38, 238.27) | 89.02 (33.41, 237.19) | 346.89 | 6.47 (5.2) | 88.71 (38.98) |
| 27 |  | Troponin i increased | 5 | 84.18 (34.95, 202.76) | 83.98 (34.76, 202.87) | 408.61 | 6.39 (5.23) | 83.7 (40.11) |
| 28 |  | Electrocardiogram t wave inversion | 4 | 47.76 (17.89, 127.48) | 47.67 (17.89, 127.01) | 182.41 | 5.57 (4.3) | 47.58 (20.92) |
| 29 |  | Ejection fraction decreased | 16 | 28.15 (17.21, 46.05) | 27.95 (17.12, 45.62) | 415.34 | 4.8 (4.11) | 27.91 (18.49) |
| 30 |  | Troponin increased | 6 | 23.55 (10.56, 52.5) | 23.49 (10.52, 52.47) | 129.06 | 4.55 (3.48) | 23.46 (12) |
| 31 | Injury, poisoning and procedural complications | Wrong drug | 3 | 262.04 (83.95, 817.99) | 261.67 (83.96, 815.57) | 770.92 | 8.02 (6.59) | 258.96 (99.9) |
| 32 |  | Product appearance confusion | 3 | 95.62 (30.75, 297.37) | 95.49 (30.64, 297.62) | 279.43 | 6.57 (5.15) | 95.13 (36.81) |
| 33 |  | Product packaging confusion | 7 | 79.39 (37.76, 166.94) | 79.13 (37.57, 166.65) | 538.33 | 6.3 (5.3) | 78.88 (42.36) |
| 34 |  | Vasoplegia syndrome | 4 | 77.53 (29.03, 207.07) | 77.38 (29.04, 206.18) | 300.65 | 6.27 (5) | 77.14 (33.91) |
| 35 |  | Incorrect route of product administration | 41 | 75.68 (55.53, 103.14) | 74.23 (55.32, 99.6) | 2954.05 | 6.21 (5.77) | 74.01 (57.13) |
| 36 |  | Accidental overdose | 33 | 26.47 (18.76, 37.34) | 26.07 (18.68, 36.38) | 795.22 | 4.7 (4.21) | 26.04 (19.53) |
| 37 |  | Wrong product administered | 5 | 22.84 (9.49, 54.95) | 22.79 (9.43, 55.05) | 104.06 | 4.51 (3.35) | 22.77 (10.92) |
| 38 |  | Incorrect drug administration rate | 3 | 20.62 (6.64, 64.01) | 20.59 (6.61, 64.17) | 55.87 | 4.36 (2.95) | 20.57 (7.97) |
| 39 | Vascular disorders | Extremity necrosis | 3 | 47.33 (15.23, 147.02) | 47.26 (15.16, 147.3) | 135.59 | 5.56 (4.14) | 47.17 (18.27) |
| 40 |  | Haemodynamic instability | 11 | 42.09 (23.26, 76.16) | 41.88 (23.26, 75.4) | 438.21 | 5.39 (4.57) | 41.81 (25.45) |
| 41 |  | Peripheral ischaemia | 3 | 20.13 (6.49, 62.51) | 20.11 (6.45, 62.68) | 54.43 | 4.33 (2.91) | 20.09 (7.79) |
| 42 |  | Ischaemia | 3 | 17.16 (5.53, 53.28) | 17.14 (5.5, 53.42) | 45.57 | 4.1 (2.68) | 17.13 (6.64) |
| 43 | Respiratory, thoracic and mediastinal disorders | Respiratory acidosis | 4 | 28.32 (10.61, 75.58) | 28.27 (10.61, 75.32) | 105.12 | 4.82 (3.55) | 28.24 (12.42) |
| 44 |  | Acute pulmonary oedema | 5 | 24.13 (10.03, 58.07) | 24.08 (9.97, 58.17) | 110.51 | 4.59 (3.43) | 24.06 (11.54) |
| 45 |  | Tachypnoea | 11 | 22.77 (12.59, 41.19) | 22.66 (12.59, 40.8) | 227.56 | 4.5 (3.68) | 22.64 (13.79) |
| 46 | General disorders and administration site conditions | Vascular stent thrombosis | 8 | 110.04 (54.88, 220.67) | 109.63 (55.21, 217.7) | 857.37 | 6.77 (5.82) | 109.15 (60.98) |
| 47 |  | Therapeutic product ineffective | 3 | 19.01 (6.12, 59.02) | 18.99 (6.09, 59.19) | 51.08 | 4.25 (2.83) | 18.97 (7.35) |
| 48 | Nervous system disorders | Tunnel vision | 3 | 44.77 (14.41, 139.08) | 44.71 (14.34, 139.35) | 127.97 | 5.48 (4.06) | 44.63 (17.29) |
| 49 | Infections and infestations | Gangrene | 5 | 22 (9.14, 52.93) | 21.95 (9.09, 53.03) | 99.89 | 4.45 (3.3) | 21.93 (10.52) |
| 50 | Congenital, familial and genetic disorders | Left ventricle outflow tract obstruction | 7 | 591.83 (279.35, 1253.86) | 589.87 (280.09, 1242.28) | 4019.77 | 9.17 (8.16) | 576.22 (307.45) |

Abbreviations: ROR, reporting odds ratio; PRR, proportional reporting ratio; IC, information component; EBGM, Empirical Bayes Geometric Mean.

Table S6 Top 50 signal strength of adverse events of intramuscular epinephrine ranked by EBGM at the preferred term level in FDA adverse event reporting system database.

| Rank | System organ class | Preferred term | Case reports | ROR (95% CI) | PRR (95% CI) | χ^2^ | IC (IC025) | EBGM (EBGM05) |
| --- | --- | --- | --- | --- | --- | --- | --- | --- |
| 1 | General disorders and administration site conditions | Injection site ischaemia | 7 | 3835.93 (1679.2, 8762.7) | 3827.83 (1680.51, 8718.94) | 21573.47 | 11.59 (10.49) | 3083.73 (1544.84) |
| 2 |  | Injection site pallor | 11 | 1107.72 (600.6, 2043.04) | 1104.05 (601.33, 2027.06) | 11333.55 | 10.01 (9.17) | 1032.25 (618.5) |
| 3 |  | Injection site laceration | 26 | 797.63 (537.16, 1184.39) | 791.39 (534.75, 1171.21) | 19548.74 | 9.56 (9) | 753.82 (541.51) |
| 4 |  | Injection site coldness | 9 | 266 (137.53, 514.49) | 265.29 (136.24, 516.58) | 2330.64 | 8.03 (7.12) | 260.94 (150.25) |
| 5 |  | Injection site hypoaesthesia | 18 | 253.76 (159.09, 404.75) | 252.38 (157.67, 403.97) | 4436.49 | 7.96 (7.3) | 248.45 (168.1) |
| 6 |  | Injection site injury | 34 | 132.34 (94.27, 185.79) | 131 (93.88, 182.8) | 4350.52 | 7.02 (6.54) | 129.93 (97.82) |
| 7 |  | Vascular stent thrombosis | 9 | 78.89 (40.94, 152) | 78.68 (41.21, 150.23) | 686.84 | 6.29 (5.39) | 78.3 (45.23) |
| 8 |  | Injection site paraesthesia | 4 | 51.63 (19.34, 137.87) | 51.57 (19.35, 137.41) | 197.72 | 5.68 (4.42) | 51.41 (22.6) |
| 9 |  | Injury associated with device | 44 | 41.48 (30.79, 55.87) | 40.94 (30.51, 54.93) | 1710.62 | 5.35 (4.93) | 40.84 (31.83) |
| 10 |  | Injection site scar | 5 | 32.4 (13.46, 77.96) | 32.35 (13.39, 78.15) | 151.6 | 5.01 (3.86) | 32.29 (15.49) |
| 11 |  | Injection site discolouration | 19 | 27.96 (17.8, 43.9) | 27.8 (17.71, 43.63) | 490.14 | 4.79 (4.16) | 27.75 (19.02) |
| 12 |  | Injection site haematoma | 18 | 20.19 (12.7, 32.09) | 20.08 (12.55, 32.14) | 326.07 | 4.33 (3.68) | 20.06 (13.61) |
| 13 |  | Crepitations | 3 | 16.79 (5.41, 52.12) | 16.78 (5.38, 52.3) | 44.46 | 4.07 (2.65) | 16.76 (6.5) |
| 14 |  | Feeling jittery | 17 | 15.36 (9.53, 24.74) | 15.28 (9.55, 24.46) | 226.79 | 3.93 (3.26) | 15.27 (10.25) |
| 15 |  | Therapeutic product ineffective | 3 | 12.12 (3.91, 37.62) | 12.11 (3.89, 37.74) | 30.56 | 3.6 (2.18) | 12.1 (4.69) |
| 16 | Cardiac disorders | Kounis syndrome | 30 | 258.66 (180.04, 371.61) | 256.33 (180.13, 364.77) | 7508.86 | 7.98 (7.46) | 252.27 (186.29) |
| 17 |  | Stress cardiomyopathy | 39 | 134.08 (97.66, 184.1) | 132.52 (96.85, 181.33) | 5048.82 | 7.04 (6.59) | 131.43 (100.81) |
| 18 |  | Coronary artery thrombosis | 8 | 66.2 (33.03, 132.67) | 66.04 (33.26, 131.14) | 510.35 | 6.04 (5.09) | 65.77 (36.76) |
| 19 |  | Arteriospasm coronary | 13 | 61.58 (35.68, 106.27) | 61.34 (35.43, 106.19) | 768.68 | 5.93 (5.17) | 61.11 (38.71) |
| 20 |  | Myocardial ischaemia | 18 | 26.16 (16.46, 41.6) | 26.03 (16.26, 41.66) | 432.57 | 4.7 (4.05) | 25.99 (17.63) |
| 21 |  | Sinus tachycardia | 19 | 23.43 (14.92, 36.8) | 23.31 (14.85, 36.59) | 405.14 | 4.54 (3.91) | 23.27 (15.95) |
| 22 |  | Long qt syndrome | 3 | 21.74 (7, 67.5) | 21.72 (6.97, 67.7) | 59.23 | 4.44 (3.02) | 21.7 (8.41) |
| 23 |  | Acute myocardial infarction | 36 | 20.74 (14.93, 28.81) | 20.52 (14.71, 28.63) | 668.11 | 4.36 (3.89) | 20.5 (15.57) |
| 24 |  | Acute coronary syndrome | 8 | 16.19 (8.09, 32.42) | 16.16 (8.14, 32.09) | 113.64 | 4.01 (3.07) | 16.14 (9.03) |
| 25 | Injury, poisoning and procedural complications | Accidental exposure to product by child | 34 | 92.01 (65.57, 129.12) | 91.08 (65.27, 127.1) | 3012.05 | 6.5 (6.02) | 90.56 (68.21) |
| 26 |  | Device use issue | 55 | 63.71 (48.78, 83.21) | 62.67 (48.57, 80.86) | 3325.55 | 5.96 (5.58) | 62.43 (49.93) |
| 27 |  | Expired product administered | 99 | 54.64 (44.72, 66.75) | 53.04 (43.6, 64.52) | 5040.39 | 5.72 (5.44) | 52.86 (44.7) |
| 28 |  | Product packaging confusion | 5 | 36.11 (15.01, 86.9) | 36.06 (14.93, 87.11) | 170.05 | 5.17 (4.01) | 35.98 (17.25) |
| 29 |  | Vascular pseudoaneurysm | 3 | 30.43 (9.8, 94.49) | 30.4 (9.75, 94.75) | 85.14 | 4.92 (3.51) | 30.34 (11.76) |
| 30 |  | Expired device used | 4 | 23.97 (8.99, 63.96) | 23.95 (8.99, 63.81) | 87.82 | 4.58 (3.31) | 23.91 (10.52) |
| 31 |  | Accidental exposure to product | 104 | 21.65 (17.8, 26.32) | 21 (17.26, 25.55) | 1981.2 | 4.39 (4.11) | 20.97 (17.81) |
| 32 |  | Product label confusion | 6 | 19.62 (8.8, 43.73) | 19.59 (8.77, 43.76) | 105.7 | 4.29 (3.22) | 19.56 (10.01) |
| 33 | Investigations | Electrocardiogram st segment depression | 11 | 85.54 (47.25, 154.86) | 85.26 (47.36, 153.5) | 911.12 | 6.41 (5.59) | 84.81 (51.61) |
| 34 |  | Electrocardiogram st segment elevation | 8 | 41.58 (20.76, 83.3) | 41.49 (20.89, 82.39) | 315.28 | 5.37 (4.43) | 41.38 (23.14) |
| 35 |  | Troponin i increased | 3 | 32.17 (10.36, 99.92) | 32.14 (10.31, 100.17) | 90.35 | 5 (3.59) | 32.08 (12.43) |
| 36 |  | Electrocardiogram t wave inversion | 4 | 30.44 (11.41, 81.24) | 30.41 (11.41, 81.03) | 113.55 | 4.92 (3.66) | 30.35 (13.35) |
| 37 |  | Troponin increased | 6 | 15.01 (6.73, 33.44) | 14.98 (6.71, 33.46) | 78.23 | 3.9 (2.83) | 14.97 (7.66) |
| 38 | Infections and infestations | Gas gangrene | 7 | 362.35 (171.16, 767.08) | 361.59 (171.69, 761.52) | 2461.03 | 8.47 (7.45) | 353.55 (188.76) |
| 39 |  | Clostridial infection | 8 | 38.48 (19.21, 77.07) | 38.39 (19.33, 76.23) | 290.61 | 5.26 (4.31) | 38.3 (21.42) |
| 40 |  | Injection site infection | 3 | 15.92 (5.13, 49.41) | 15.91 (5.1, 49.59) | 41.87 | 3.99 (2.57) | 15.89 (6.16) |
| 41 |  | Necrotising fasciitis | 3 | 15.43 (4.97, 47.89) | 15.42 (4.95, 48.06) | 40.41 | 3.95 (2.53) | 15.4 (5.97) |
| 42 | Vascular disorders | Vasoconstriction | 8 | 179.11 (89.15, 359.86) | 178.68 (88.23, 361.84) | 1397.78 | 7.47 (6.52) | 176.7 (98.56) |
| 43 |  | Vasospasm | 3 | 58.28 (18.75, 181.18) | 58.23 (18.68, 181.49) | 168.13 | 5.86 (4.44) | 58.02 (22.46) |
| 44 |  | Poor peripheral circulation | 5 | 20.02 (8.32, 48.16) | 19.99 (8.27, 48.29) | 90.11 | 4.32 (3.16) | 19.97 (9.58) |
| 45 | Nervous system disorders | Cerebral vasoconstriction | 3 | 68.02 (21.87, 211.54) | 67.96 (21.8, 211.82) | 197.09 | 6.08 (4.66) | 67.68 (26.19) |
| 46 |  | Reversible cerebral vasoconstriction syndrome | 3 | 30.46 (9.81, 94.61) | 30.44 (9.77, 94.87) | 85.25 | 4.93 (3.51) | 30.38 (11.77) |
| 47 |  | Sensory loss | 5 | 10.78 (4.48, 25.92) | 10.76 (4.45, 25.99) | 44.25 | 3.43 (2.27) | 10.75 (5.16) |
| 48 | Respiratory, thoracic and mediastinal disorders | Hyperventilation | 4 | 11.84 (4.44, 31.57) | 11.82 (4.44, 31.49) | 39.61 | 3.56 (2.3) | 11.82 (5.2) |
| 49 | Musculoskeletal and connective tissue disorders | Necrotising myositis | 3 | 72.7 (23.37, 226.11) | 72.63 (23.3, 226.37) | 210.98 | 6.18 (4.76) | 72.31 (27.98) |
| 50 | Immune system disorders | Anaphylactic reaction | 53 | 17.93 (13.66, 23.52) | 17.66 (13.42, 23.24) | 832.58 | 4.14 (3.75) | 17.64 (14.05) |

Abbreviations: ROR, reporting odds ratio; PRR, proportional reporting ratio; IC, information component; EBGM, Empirical Bayes Geometric Mean.
